# Checkpoint inhibitors as dual immunotherapy in advanced non-small cell lung cancer: a meta-analysis

**DOI:** 10.3389/fonc.2023.1146905

**Published:** 2023-06-15

**Authors:** Muyesar Alifu, Min Tao, Xiao Chen, Jie Chen, Kejing Tang, Yubo Tang

**Affiliations:** ^1^ Department of Pharmacy, The First Affiliated Hospital, Sun Yat-sen University, Guangzhou, Guangdong, China; ^2^ Department of Pulmonary and Critical Care Medicine, The First Affiliated Hospital, Sun Yat-sen University, Guangzhou, Guangdong, China

**Keywords:** non-small cell lung cancer (NSCLC), programmed death-1/cytotoxic antigen 4 inhibitors (PD-1/CTAL-4 inhibitors), anti-TIGIT antibodies, dual immunotherapy, meta-analysis

## Abstract

**Introduction:**

Recent clinical trials have confirmed that anti-programmed cell death-1/ligand 1 (anti-PD-1/L1) combined with either anti-cytotoxic T-lymphocyte-associated protein 4 (anti-CTLA-4) or anti-T-cell immunoreceptor with Ig and ITIM domains (TIGIT) antibodies (dual immunotherapy) produced significant benefits as first-line therapies for patients with advanced non-small cell lung cancer (NSCLC). However, it also increased the incidence of adverse reactions, which cannot be ignored. Our study aims to explore the efficacy and safety of dual immunotherapies in advanced NSCLC.

**Methods:**

This meta-analysis ultimately included nine first-line randomized controlled trials collected from PubMed, EMBASE, and Cochrane Central Register of Controlled Trials databases until 13 August 2022. Efficacy was measured as the hazard ratio (HR) and 95% confidence interval (CI) for progression-free survival (PFS), overall survival (OS), and risk ratio (RR) for the objective response rates (ORRs). Treatment safety was assessed by RR of any grade of treatment-related adverse events (TRAEs) and grade ≥ 3 TRAEs.

**Results:**

Our results demonstrated that, compared to chemotherapy, dual immunotherapy shows durable benefits in OS (HR = 0.76, 95% CI: 0.69–0.82) and PFS (HR = 0.75, 95% CI: 0.67–0.83) across all levels of PD-L1 expression. Subgroup analysis also presented that dual immunotherapy resulted in improved long-term survival compared with chemotherapy in patients with a high tumor mutational burden (TMB) (OS: HR = 0.76, *p* = 0.0009; PFS: HR = 0.72, *p* < 0.0001) and squamous cell histology (OS: HR = 0.64, *p* < 0.00001; PFS: HR = 0.66, *p* < 0.001). However, compared with immune checkpoint inhibitor (ICI) monotherapy, dual immunotherapy shows some advantages in terms of OS and ORR and only improved PFS (HR = 0.77, *p* = 0.005) in PD-L1 < 25%. With regard to safety, there was no significant difference in any grade TRAEs (*p* = 0.05) and grade ≥ 3 TRAEs (*p* = 0.31) between the dual immunotherapy and chemotherapy groups. However, compared with ICI monotherapy, dual immunotherapy significantly increased the incidence of any grade TRAEs (*p* = 0.03) and grade ≥ 3 TRAEs (*p* < 0.0001).

**Conclusions:**

As for the efficacy and safety outcome, compared with standard chemotherapy, dual immunotherapy remains an effective first-line therapy for patients with advanced NSCLC, especially for patients with high TMB levels and squamous cell histology. Furthermore, compared to single-agent immunotherapy, dual immunotherapy is only considered for use in patients with low PD-L1 expression in order to reduce the emergence of resistance to immunotherapy.

**Systematic Review Registation**

https://www.crd.york.ac.uk/PROSPERO/, identifier CRD42022336614.

## 1 Introduction

Lung cancer is one of the most frequently diagnosed cancers and the leading cause of cancer-related deaths worldwide. In particular, non-small cell lung cancer (NSCLC) account for 85% of lung cancer. Within NSCLC classifications, adenocarcinomas are the most common subtype of lung cancer, followed by squamous cell carcinomas (1). Briefly, tumors that have clear morphologic patterns of adenocarcinoma (acinar, papillary, lepidic, and micropapillary) or squamous cell carcinoma (unequivocal keratinization and well-formed classical bridges) can be diagnosed as adenocarcinoma or squamous cell carcinoma (2). For many years, whether squamous cell carcinoma or non-squamous cell carcinoma, platinum-based doublet chemotherapy has been the conventional first-line therapy for patients with advanced NSCLC that is driver gene negative (3). Since 2015, four different immune checkpoint inhibitors (ICIs) have been approved by the Food and Drug Administration (FDA) for use in the management of advanced NSCLC, including anti-PD-1 antibodies nivolumab and pembrolizumab, and anti-PD-L1 antibodies atezolizumab and durvalumab (4). The expression of PD-L1 on the surface of tumor cells, detected by immunohistochemistry, is a predictive biomarker used to guide treatment decisions with anti-PD-1 or anti-PD-L1 antibodies in patients with NSCLC (1). The PD-L1 expression level of the tumor was recorded as a percentage of PD-L1-positive tumor cells over the total tumor cells: tumor proportion score (TPS) (5). A study confirms that PD-L1-positive (TPS ≥ 1%) lung cancer was more frequent in squamous cell carcinoma than adenocarcinoma (5). Compared with chemotherapy, PD-1/L1 checkpoint inhibitors significantly improved long-term survival in patients with PD-L1 positive or high expression (6–10), which elevated the status of immunotherapeutic from secondary to first-line. For an anticancer immune response to lead to effective killing of cancer cells, a series of stepwise events must be initiated and allowed to proceed and expand iteratively. We refer to these steps as the cancer-immunity cycle (11). However, there are still some patients who cannot respond to immunotherapy; PD-L1 blockade is also limited by a low response rate in some cancers, lack of known biomarkers, immune-related toxicity, and resistance to both innate and acquired drugs, with the most recent data clarifying that the clinical response to PD-1/PD-L1 blockade is barely 40% (12). Therefore, this is a strong call to explore new therapeutic drugs or combination therapy strategies to increase the clinical utilization of immune checkpoint inhibitors.

Recent studies show that the combination of anti-CTLA-4 or anti-PD-1/L1 (dual immunotherapy) with the blockade of other immune checkpoints or with the activation of co-stimulatory molecules can also further amplify anti-tumor immune responses (13). In addition, combination therapy revealed the greatest benefit in patients who were less likely to benefit from PD-L1 inhibition or PD-1 inhibition alone, particularly with negative expression of PD-L1. The addition of a CTLA-4-targeted therapy may be completing the defect in the cancer-immunity cycle for patients who are PD-L1-negative (11). The results of CheckMate 227 study part 1 showed that compared with chemotherapy, the combination of nivolumab (PD-1 inhibitor) and ipilimumab (CTLA-4 inhibitor) resulted in significant overall survival (OS) benefit in patients with PD-L1 ≥ 1% (median, 17.1 vs 14.9 months, HR = 0.76), the rate of CR improved to 5.8%, the median Duration of Overall Response (DOR) was 23.2 months, and the OS was also beneficial in patients with PD-L1 TPS < 1% (median, 17.2 vs 12.2 months HR = 0.64) (14). Tiragolumab is a fully human IgG1-kappa anti-TIGIT monoclonal antibody with an intact region of Fc that blocks TIGIT binding to the PVR protein. Blocking negative regulation with an anti-TIGIT antibody may restore the anti-tumor immune response (15). The phase 2 CITYSCAPE trial demonstrates that tiragolumab (TIGIT inhibitor) plus atezolizumab (PD-L1 inhibitor) resulted in a clinically significant improvement in the objective response rate (31.3% vs 16.2%) and progression-free survival (median, 5.4 vs 3.6 months, HR = 0.57) compared with placebo plus atezolizumab in patients with chemotherapy-naive, PD-L1-positive, recurrent, or metastatic NSCLC (16).

However, some clinical trials fail to achieve the anticipated clinical efficacy of dual immunotherapy and had to suspend clinical research due to the increased incidence of high-frequency adverse events. A randomized, double-blind phase III KEYNOTE-598 trial shows that the combination of ipilimumab (CTLA-4 inhibitor) and pembrolizumab (PD-1 inhibitor) cannot improve efficacy and is associated with greater toxicity than pembrolizumab monotherapy as a first-line treatment for metastatic NSCLC with PD-L1 TPS ≥ 50% and no targetable EGFR or ALK aberrations (17).

In summary, dual immunotherapy (anti-PD-1/L1 antibody plus anti-CTLA-4 antibody or TIGIT antibody) is controversial as a first-line therapy for advanced non-small cell lung cancer. We performed this meta-analysis to evaluate the efficacy and safety of dual immunotherapy versus chemotherapy or checkpoint inhibitor monotherapy in patients with advanced NSCLC and to select patient characteristics that may be more suitable for dual immunotherapy treatment strategies.

## 2 Methods

### 2.1 Search strategy

We searched eligible phase II and III randomized controlled trials (RCTs) from PubMed, EMBASE, and Cochrane Central Register of Controlled Trials databases by using the following keywords: advanced/metastatic non-small cell lung cancer, checkpoint inhibitors, PD-1, PD-L1, cytotoxic T-lymphocyte associated protein 4, TIGIT antibody, nivolumab, pembrolizumab, durvalumab, ipilimumab, tremelimumab, and randomized/controlled clinical trial (more search details are shown in **Supplementary Table 1**). To make sure all relevant references were involved, the searched keywords were MeSH terms combined with logical operator. The RCTs conducted until 13 August 2022 and published in English with no country restrictions were searched. Our systematic review strategy was submitted to the PROSPERO website and was given the registration number CRD42022336614.

### 2.2 Data extraction and quality assessment of the included studies

Across all studies included in this meta-analysis, the following are the inclusion criteria: (S) type of literature: phase II/III RCTs. (P) The patients enrolled in the study were adults with stage III/VI NSCLC and Eastern Cooperative Oncology Group (ECOG) performance status of 0 or 1. (I) Patients in the intervention group were treated with anti-PD-1/L1 antibody plus anti-CTLA-4 antibody or anti-TIGIT antibody. (C) The control group received chemotherapy or anti-PD-1/L1 inhibitor therapy only. (O) Outcome measures included the objective response rate (ORR), as well as the hazard ratio (HR) of progression-free survival (PFS) and overall survival (OS), along with their 95% confidence intervals (95% CIs) between the experimental arm and the control group. Data are available. The exclusion criteria were as follows: 1) non-randomized controlled trials, 2) sensitizing EGFR mutations or known translocations of ALK, 3) patients with known or suspected active autoimmune disease, and 4) papers with the same research population.

Data extraction was performed independently by two authors (Muyesar Alifu and Tao Ming) according to the inclusion criteria. Disagreements were resolved by discussion until a consensus was reached or resolved by a third author. Extracted data included 1) author, year of publication, stage of treatment, intervention, medication, follow-up time, and sample size; 2) patient gender, age, histological type of tumor, and PD-1/L1 expression and ECOG performance status; 3) the primary outcome was OS and PFS; 4) the additional outcomes include ORR for efficacy and all grades of treatment-related adverse events (TRAEs) or grade (G) ≥ 3 TRAEs. Cochrane Collaboration’s tool was used to assess the risk of bias in each eligible randomized trial (18).

Two independent reviewers assessed the study quality using the following criteria: random sequence (selection bias), allocation concealment (selection bias), blinding of participants and researchers (performance bias), blinding of outcome assessment (detection bias), incomplete outcome data (attrition bias), selective reporting (reporting bias), and other sources of bias (18). For each study, we defined “yes” as a low risk of bias and “no” as a high risk of bias. We defined also “unclear” if there were insufficient data for a precise judgment (19).

All clinical outcomes were measured by Response Evaluation Criteria in Solid Tumors (version 1.1). ORR was defined as the proportion of patients with the best overall response or partial response or better. PFS was defined as the time from randomization to the date of the first documented tumor progression, or death from any cause, whichever occurred first. OS was defined as the time from randomization to the date of death from any cause. Complete response (CR) was defined as the disappearance of all target lesions and any pathological lymph nodes (whether target or non-target) that must have a reduction in the short axis to <10 mm. Partial response (PR) was defined as at least a 30% decrease in the sum of diameters of target lesions, taking as reference the baseline sum diameters (20). Confirmation of response was required at least 4 weeks after the initial response. The assessment of adverse events was according to National Cancer Institute Common Terminology Criteria for Adverse Events (version 4.0).

### 2.3 Statistical analysis

A comparison of efficacy between dual immunotherapy and other treatments was conducted by HR and 95% confidence interval (CI) of the PFS and OS, and risk ratio (RR) of the ORR. Safety of treatment was assessed by RR of all TRAEs and grade ≥ 3 treatment-related adverse events (G ≥ 3 TRAEs), TRAEs leading to discontinuation, TRAEs leading to death, immune-related adverse events (irAEs), and grade ≥ 3 immune-related adverse events (G ≥ 3 irAEs). Heterogeneity among treatment groups was assessed by the chi-square-based Q statistic. If *I* ^2^ > 50% or *p* < 0.05, a random-effects model was adopted. Otherwise, a fixed-effects model was employed. *p* < 0.05 is considered statistically significant in the whole statistical test.

To explore the sources of heterogeneity, we performed subgroup analyses of the following: level of PD-L1 expression, tumor mutational burden, age, gender, ECOG performance status, smoking status, histologic characteristics, etc. For all the studies, we used Egger’s test providing the funnel plot for the evaluation of publication bias (**Supplementary Figure 1**). It should be noted that the articles included in this meta-analysis were based on the latest or most complete follow-up data, and all data analyses were carried out using Review Manager 5.4 and Stata (version 16.0). Preferred Reporting Items for Systematic Reviews and Meta-Analyses (PRISMA) statements were used as the basis for the study design of this meta-analysis (21).

## 3 Results

Our initial search yielded a total of 383 studies. Following abstract screening and full-text reviewing, we identified nine clinical trials eligible for inclusion in the meta-analysis (14, 16, 17, 22–27). It is important to note that two of the included studies (NEPTUNE (27) and POSEIDON (26)) were retrieved from searches of the Cochrane Central Register of Controlled Trials databases and have yet to be published. **Supplementary Figure 2** provides details of the selection process and reasons for exclusion.

Of the included studies, a total of 2,792 patients received dual immunotherapy, 2,263 patients received chemotherapy, 656 patients received ICI monotherapy, and 507 patients received ICI combined chemotherapy. Eight studies investigated anti-PD-1/L1 antibodies combined with anti-CTLA-4 antibodies, and one study (CITYSCAPE) investigated anti-PD-L1 antibodies combined with anti-TIGIT antibodies. Seven studies explored dual immunotherapy as a first-line treatment strategy, whereas two studies explored dual immunotherapy as a second-line or later treatment strategy. All involved studies measured tumor cell/tissue PD-L1 expression. The results of all clinical trials are the most recently published. Notably, CheckMate 227 used the latest 4 years’ outcome (14), CheckMate-9LA used the latest 2 years’ update (25), and KEYNOTE-598 used the latest 3 years’ follow-up data (28). Baseline characteristics and outcome data of eligible studies are summarized in **Table 1** and **Supplementary Tables 2, 3**.

**Table 1:** Main baseline characteristics of each included trial considered in this meta-analysis.

	Treatment arm	Control arm	Number of patients	Median age	Male (%)	Squamous (%)
CheckMate-227 part 1, 2022 (14)	Nivolumab + ipilimumab	Chemotherapy	583 vs 583	64 vs 64	67.4 vs 66	28 vs 27.8
CheckMate-9LA, 2021 (25)	Nivolumab + ipilimumab + chemotherapy	Chemotherapy	361 vs 358	65 vs 65	70 vs 70	31 vs 31
MYSTIC, 2020 (23)	Durvalumab + tremelimumab	Chemotherapy	372 vs 372	66 vs 64	71.5 vs 67.2	28.8 vs 28.5
	Durvalumab + tremelimumab	Durvalumab (BTMB > 20)	64 vs 77	66 vs 67	73.4 vs 75.3	39.1 vs 37.7
	Durvalumab + tremelimumab	Durvalumab (BTMB < 20)	204 vs 209	65 vs 64	69.6 vs 68.9	27.9 vs 28.2
The Lung-MAP, 2021 (24)	Nivolumab + ipilimumab	Nivolumab	125 vs 127	67 vs 68	66 vs 68	100 vs 100
KEYNOTE-598, 2022 (17)	Pembrolizumab + ipilimumab	Pembrolizumab-placebo	284 vs 284	64 vs 65	71.1 vs 67.3	27.1 vs 28.5
ARCTIC, 2020 (22)	Durvalumab + tremelimumab	Soc	174 vs 118	63 vs 65	66.1 vs 68.6	24.1 vs 23.7
	Durvalumab + tremelimumab	Durvalumab	174 vs 117	63 vs 63	66.1 vs 62.4	24.1 vs 24.8
	Durvalumab + tremelimumab	Tremelimumab	174 vs 60	63 vs 64	66.1 vs 65	24.1 vs 25
CITYSCAPE, 2022 (16)	Tiragolumab + atezolizumab	Atezolizumab-placebo	67 vs 68	68 vs 68	58 vs 71	40 vs 41
NEPTUNE, 2022 (27)	Durvalumab + tremelimumab	Soc (global)^a^	410 vs 413	NR	72.4 vs 73.8	NR
	Durvalumab + tremelimumab	Soc (China)^b^	78 vs 82	NR	76.9 vs 69.5	NR
POSEIDON, 2022 (26)	Durvalumab + tremelimumab + Soc	Soc	338 vs 337	63 vs 63	79.6 vs 73.6	NR
	Durvalumab + tremelimumab + Soc	Durvalumab + Soc	338 vs 338	63 vs 64	79.6 vs 74.9	NR

Anti-PD-1 inhibitor: nivolumab and pembrolizumab. Anti-PD-L1 inhibitor: durvalumab and atezolizumab. Anti-CTLA-4 inhibitor: ipilimumab and tremelimumab. Anti-TIGIT inhibitor: tiragolumab.

Soc, standard of care chemotherapy; BTMB, blood tumor mutational burden; NR, not reported.

a Participants in global cohort.

b Participants in China cohort.

### 3.1 Benefit of dual immunotherapy

**Figure 1:**
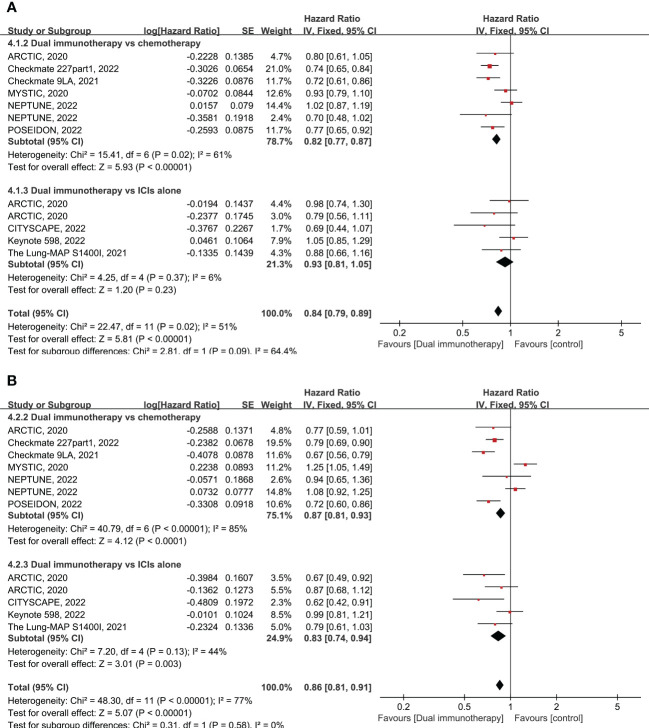
Forest plot of hazard ratio (HR). Comparison of overall survival (OS) **(A)** and progression-free survival (PFS) **(B)** between dual immunotherapy and other treatments. ICIs, immune checkpoint inhibitors.

Nine RCTs enrolling 5,711 patients evaluated the OS of dual immunotherapy versus chemotherapy or ICI monotherapy. OS was greater in patients treated with dual immunotherapy. As shown in the pooled result, patients treated with dual immunotherapy had a significantly prolonged OS compared with patients treated with either chemotherapy or ICI monotherapy (pooled HR = 0.84 95% CI: 0.79–0.89, *p* < 0.00001, *I* ^2 ^= 51%) (**Figure 1A**). Subgroup analysis showed that dual immunotherapy produced a large benefit compared with chemotherapy (pooled HR = 0.82, 95% CI: 0.77–0.97, *p* < 0.00001, *I* ^2 ^= 61%), but there were no clear benefits in OS between dual immunotherapy and ICI monotherapy (pooled HR = 0.93, 95% CI: 0.81–1.05, *p* = 0.23, *I* ^2 ^= 6%) (**Figure 1A**). No dissymmetry was observed in the funnel plot for the OS (**Supplementary Figure 1A**; Egger’s test, *p* = 0.969) test, highlighting that there was no obvious publication bias with respect to OS.

Nine RCTs enrolling 5,690 patients evaluated the PFS of dual immunotherapy versus chemotherapy or ICI monotherapy. The PFS was significantly higher in patients treated with dual immunotherapy (pooled HR = 0.86 95% CI: 0.81–0.91, *p* < 0.00001, *I* ^2 ^= 77%) compared to that of patients treated with either chemotherapy alone or ICIs alone (**Figure 1B**). Subgroup analysis shows that dual immunotherapy is associated with a slight advantage over chemotherapy (pooled HR = 0.87, 95% CI: 0.81–0.93, *p* < 0.0001, *I* ^2 ^= 85%) as well as large improved PFS when compared with ICI monotherapy (pooled HR = 0.83, 95% CI: 0.74–0.94, *p* = 0.003, *I* ^2 ^= 44%) (**Figure 1B**). No dissymmetry was observed in the funnel plot for the PFS (**Supplementary Figure 1B**; Egger’s test, *p* = 0.494) test, highlighting that there was no obvious publication bias regarding the PFS.

Nine RCTs enrolling 6,218 patients evaluated the ORR of dual immunotherapy versus chemotherapy or ICI monotherapy or ICIs + chemotherapy. There was a significantly higher ORR in patients who were treated with dual immunotherapy when compared with other therapies (pooled RR = 1.08, 95% CI: 1.01–1.15, *p* = 0.03, *I* ^2 ^= 79%) (**Supplementary Figure 3**). Note that subgroup analysis shows that dual immunotherapy had a greater advantage over ICI monotherapy (pooled RR = 1.20, 95% CI: 1.05–1.38, *p* = 0.01, *I* ^2 ^= 28%). In contrast, there was no significant difference between dual immunotherapy and either chemotherapy (pooled RR = 1.09, 95% CI: 1.00–1.19, *p* = 0.05, *I* ^2 ^= 88%) or ICIs + chemotherapy (pooled RR = 0.88, 95% CI: 0.77–1.02, *p* = 0.09, *I* ^2 ^= 62%) (**Supplementary Figure 3**). No dissymmetry was observed in the funnel plot for the ORR (**Supplementary Figure 1C**; Egger’s test, *p* = 0.446), pointing out that there was no obvious publication bias regarding ORR.

#### 3.1.1 Subgroup analyses by PD-L1 expression level

Given that PD-L1 expression was adopted as the dominant biomarker for screening patients for treatment with ICIs, in this study, we compared the efficacy of dual immunotherapy with chemotherapy or ICI monotherapy in different PD-L1 expression levels.

**Figure 2:**
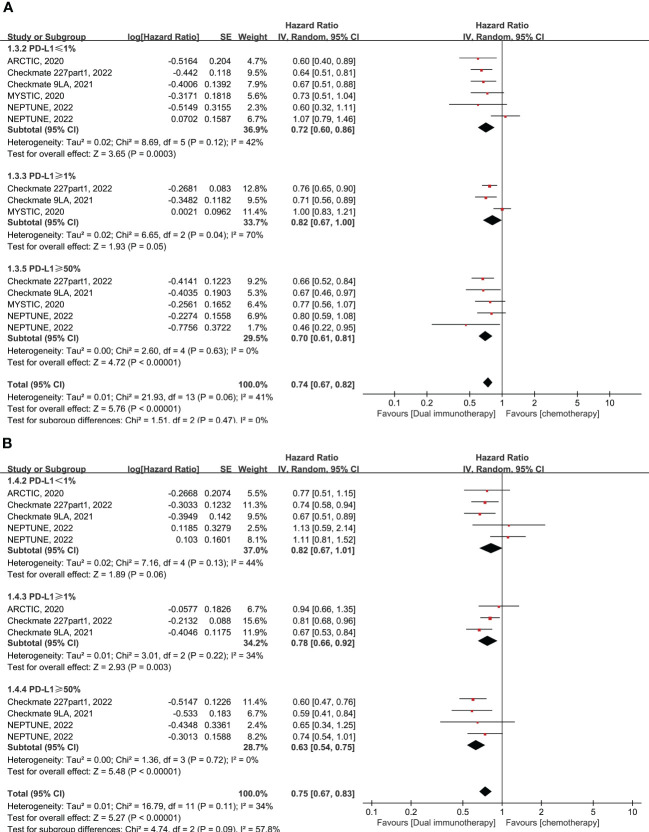
Forest plot of hazard ratio (HR). Comparison of overall survival (OS) **(A)** and progression-free survival (PFS) **(B)** between dual immunotherapy and chemotherapy depending on PD-L1 expression level.

A total of five RCTs enrolling 1,966 patients evaluated dual immunotherapy versus chemotherapy in advanced NSCLC. In comparison to chemotherapy, dual immunotherapy shows significant advantages in terms of OS (pooled HR = 0.74, 95% CI: 0.67–0.82, *p* < 0.00001, *I* ^2 ^= 41%) and PFS (pooled HR = 0.75, 95% CI: 0.67–0.83, *p* < 0.00001, *I* ^2 ^= 34%) in all subgroups of PD-L1 expression levels that have been tested, particularly in the PD-L1 expression of less than 1% (pooled HR = 0.72 for OS and HR = 0.82 for PFS) (**Figure 2**). The most obvious improvement in long-term clinical efficacy is shown in patients with PD-L1 ≥ 50% (pooled HR = 0.70, 95% CI: 0.61–0.81, *p* < 0.00001, *I* ^2 ^= 0% for OS; pooled HR = 0.63, 95% CI: 0.54–0.75, *p* < 0.00001, *I* ^2 ^= 0% for PFS) (**Figure 2**). There was no significant interaction between treatment effect in terms of ORR and PD-L1 expression level in the comparison of dual immunotherapy and chemotherapy (pooled RR = 1.05, 95% CI: 0.87–1.26, *p* = 0.61, *I* ^2 ^= 64%) (**Supplementary Figure 4A**).

**Figure 3:**
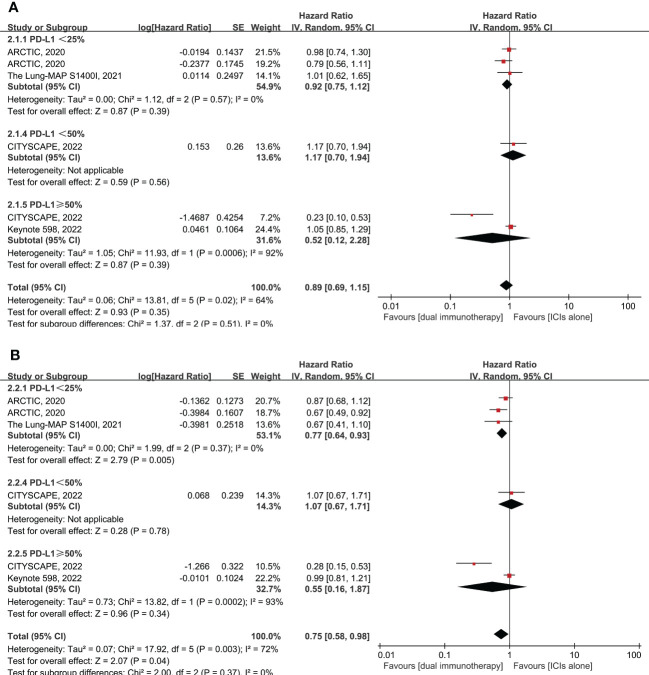
Forest plot of hazard ratio (HR). Comparison of overall survival (OS) **(A)** and progression-free survival (PFS) **(B)** between dual immunotherapy and ICI monotherapy depending on PD-L1 expression level. ICIs, immune checkpoint inhibitors.

Six RCTs enrolling 1,801 patients evaluated dual immunotherapy versus ICI monotherapy in advanced NSCLC. The PFS was higher in patients who were treated with dual immunotherapy compared to ICI monotherapy, with a pooled HR of 0.75 (95% CI: 0.58–0.98, *p* = 0.04, *I* ^2 ^= 72%) (**Figure 3**). Furthermore, the benefit of PFS is more reflected in the PD-L1 < 25% subgroup, with pooled HR of 0.77 (95% CI: 0.64–0.93, *p* = 0.005, *I* ^2 ^= 0%), rather than in the PD-L1 ≥ 50% subgroup, with pooled HR of 0.55 (95% CI: 0.16–1.87, *p* = 0.34, *I* ^2 ^= 93%) (**Figure 3**). However, in comparison to monotherapy with ICIs, dual immunotherapy does not have a significant benefit in terms of OS (pooled HR = 0.89, 95% CI: 0.69–1.15, *p* = 0.35, *I* ^2 ^= 64%) (**Figure 3**) and ORR (pooled RR = 1.14, 95% CI: 0.82–1.59, *p* = 0.43, *I* ^2 ^= 53%) (**Supplementary Figure 4B**) among different levels of PD-L1 expression. In summary, dual immunotherapy demonstrates little clinical benefit when compared with ICI monotherapy, but we cannot ignore the fact that the CITYSCAPE clinical trial shows very impressive clinical efficacy in patients with PD-L1 ≥ 50% who were treated with anti-PD-L1 plus anti-TIGIT antibodies (pooled HR = 0.23 for OS and HR = 0.28 for PFS) (**Figure 3**).

#### 3.1.2 Subgroup analyses by tumor mutational burden

Tumor mutational burden is an emerging and independent biomarker of outcome with immunotherapy in multiple tumor types, including lung cancer (29–31). Our meta-analysis included four RCTs enrolling 2,256 patients that evaluated dual immunotherapy versus alternative therapy on the basis of tumor mutational burden (TMB) in advanced NSCLC. Due to limited data, the TMB result in the study includes both tissue and blood tests.

In patients with a high tumor mutational burden (determined as at least 10 mutations per megabats), dual immunotherapy led to a significantly increased in OS (pooled HR = 0.76, 95% CI: 0.64–0.89, *p* = 0.0009, *I* ^2 ^= 15%) and PFS (pooled HR = 0.72, 95% CI: 0.62–0.84, *p* < 0.0001, *I* ^2 ^= 6%) when compared to other treatments (**Supplementary Figures 5A, B**). The therapeutic advantages of dual immunotherapy are more reflected in comparison with chemotherapy. In the high TMB subgroup, dual immunotherapy produced significant advantages in terms of OS (pooled HR = 0.69, 95% CI: 0.55–0.88, *p* = 0.003, *I* ^2 ^= 33%) and PFS (pooled HR = 0.71, 95% CI: 0.57–0.88, *p* = 0.002, *I* ^2 ^= 36%) over chemotherapy (**Supplementary Figures 5A, B**). It should be noted that the ORR was not significantly different between dual immunotherapy and chemotherapy in patients with a high level of TMB (pooled RR = 1.21, 95% CI: 0.72–2.05, *p* = 0.48, *I* ^2 ^= 84%) (**Supplementary Figure 6**). Overall, compared with ICI monotherapy, dual immunotherapy in patients with high TMB expression had a modest advantage in terms of OS (pooled HR = 0.85, 95% CI: 0.67–1.09, *p* = 0.21, *I* ^2 ^= 0%) and PFS (pooled HR = 0.76, 95% CI: 0.57–1.00, *p* = 0.05, *I* ^2 ^= 0%) with no statistical significance (**Supplementary Figures 5A, B**).

On the contrary, for patients with a low tumor mutational burden (determined as TMB <10 or 20 mut/Mb), there was a substantial benefit in terms of both PFS (pooled HR = 1.32, 95% CI: 1.05–1.65, *p* = 0.02, *I* ^2 ^= 71%) and OS (pooled HR = 1.15, 95% CI: 1.04–1.29, *p* = 0.009. *I* ^2 ^= 0%) observed in favor of chemotherapy or ICI monotherapy (**Supplementary Figures 5C, D**).

#### 3.1.3 Subgroup analysis by tumor histology type

We sought to explore the efficacy of dual immunotherapy in different histology types by exploring the therapeutic efficacy of dual immunotherapy and other monotherapies in patients with both squamous and non-squamous NSCLC. Six RCTs enrolling 3,132 patients evaluated the clinical efficacy of dual immunotherapy versus chemotherapy or ICI monotherapy in different histology types.

In comparison to chemotherapy, dual immunotherapy resulted in a significant increase in OS (pooled HR = 0.64, 95% CI: 0.54–0.77, *p* < 0.00001, *I* ^2 ^= 0% for squamous and pooled HR = 0.75, 95% CI: 0.66–0.84, *p* < 0.00001, *I* ^2 ^= 0% for non-squamous) and PFS (pooled HR = 0.66, 95% CI: 0.54–0.80, *p* < 0.0001, *I* ^2 ^= 0% for squamous and pooled HR = 0.78, 95% CI: 0.69–0.89, *p* = 0.0002, *I* ^2 ^= 4% for non-squamous) in both squamous and non-squamous patients (**Supplementary Figures S7, 8**). The benefit of dual immunotherapy on ORR was only seen in patients with squamous histology type when compared with chemotherapy (pooled RR = 1.49, 95% CI: 1.17–1.88, *p* = 0.001, *I* ^2 ^= 0%) (**Supplementary Figure 9**).

However, regardless of histologic type, there was no significant difference between dual immunotherapy and ICI monotherapy in terms of OS and PFS (**Supplementary Figures S7, 8**), as it was only in non-squamous patients (pooled RR = 1.34, 95% CI: 1.07–1.69, *p* = 0.01, *I* ^2 ^= 0%) that the advantage of dual immunotherapy in terms of the response rate emerges (**Supplementary Figure 9**).

#### 3.1.4 Subgroup analysis by other factors

The number of trials with available data of OS subgroup analysis between dual immunotherapy and chemotherapy is summarized in **Table 2**. Overall, there were some factors that might predict OS benefit from dual immunotherapy compared with chemotherapy. The following might acquire more OS advantages from dual immunotherapy: both male and female patients (male, pooled HR = 0.68, vs female, pooled HR = 0.80), with greater benefit in male patients (interaction, *p* < 0.00001); with younger age (<65 years, pooled HR = 0.69, vs ≥75 years, pooled HR = 0.96; interaction, *p* < 0.00001); in both ECOG PS = 0 and ECOG PS = 1 (PS = 0, pooled HR = 0.59, vs PS = 1, pooled HR = 0.76) and with greater benefit seen in patients with ECOG PS = 0 (interaction, *p* < 0.00001); smokers (smoker, pooled HR = 0.69, vs never-smoker, pooled HR = 1.00; interaction, *p* < 0.00001); no liver metastases (yes, pooled HR = 0.82, vs no, pooled HR = 0.69; interaction, *p* < 0.00001); in patients with and without bone metastases (yes, pooled HR = 0.70, vs no, pooled HR = 0.72 interaction, *p* < 0.00001); in patients with and without central nervous system (CNS) metastases (yes, pooled HR = 0.51, vs no, pooled HR = 0.74) and with a greater benefit in patients with CNS metastases (interaction, *p* = 0.0001); and with an anti-PD-1/L1 antibody (anti-PD-1, pooled HR = 0.73, vs anti-PD-L1, pooled HR = 0.87) and with a greater benefit with PD-1 antibody (interaction, *p* = 0.0004). Due to the limited data available, we did not analyze the OS subgroup between dual immunotherapy and ICI monotherapy.

**Table 2:** Summary of OS hazard ratios in subgroup analysis comparing overall survival in patients who received dual immunotherapy vs chemotherapy.

Overall survival						
Group	No. of studies	No of patients	HR (95% CI)	*p*	Interaction, *p*	*I* ^2^ (%)
Sex						
Male	3	1,478	0.68 (0.61, 0.77)	<0.00001	<0.00001	0%
Female	3	649	0.80 (0.67, 0.97)	0.02		0%
Age						
<65 years	3	1,117	0.69 (0.60, 0.79)	<0.00001	<0.00001	0%
65–75 years	3	877	0.72 (0.62, 0.84	<0.0001		0%
≥75 years	2	183	0.96 (0.69, 1.34)	0.81		1%
ECOG PS						
0	2	620	0.59 (0.42, 0.84)	0.003	<0.00001	57%
1	2	1,255	0.76 (0.67, 0.86)	<0.0001		0%
Smoking status						
Never-smoker	3	311	1.00 (0.76, 1.31)	0.99	<0.00001	0%
Smoker	3	1,853	0.69 (0.62, 0.77)	<0.00001		0%
Histology type						
Squamous	3	625	0.64 (0.54, 0.77)	<0.00001	<0.00001	0%
Non-squamous	3	1,552	0.75 (0.66, 0.84)	<0.00001		0%
Liver metastasis						
Yes	2	406	0.82 (0.66, 1.02)	0.08	<0.00001	0%
No	2	1,479	0.69 (0.61, 0.78)	<0.00001		0%
Bone metastasis						
Yes	2	523	0.70 (0.57, 0.85)	0.0003	<0.00001	0%
No	2	1,362	0.72 (0.63, 0.81)	<0.00001		0%
CNS metastasis						
Yes	2	237	0.51 (0.29, 0.89)	0.02	0.0001	70%
No	2	1,648	0.74 (0.66, 0.83)	<0.00001		0%
IO drug						
Anti-PD-1	2	1,885	0.73 (0.66, 0.81)	<0.00001	0.0004	0%
Anti-PD-L1	4	2,673	0.87 (0.76, 0.99)	0.03		50%

ECOG PS, Eastern Cooperative Oncology Group performance status; IO, immuno-oncology; CNS, central nervous system.

### 3.2 Safety analysis

As for safety, we mainly evaluated any grade treatment-related adverse events, grade 3–4 adverse events, and AEs leading to treatment discontinuation. The number of patients included in each safety analysis is presented in **Supplementary Tables 3, 4**.

In general, there was no significant difference in any grade TRAEs between dual immunotherapy and any other therapy (pooled RR = 0.96, 95% CI: 0.89–1.03, *p* = 0.25, *I* ^2 ^= 88%). Subgroup analysis revealed that there were no significant differences between dual immunotherapy and chemotherapy in terms of the frequency of any grade TRAEs (RR = 0.89, 95% CI: 0.79–1.00, *p* = 0.05, *I* ^2 ^= 92%). However, when compared with ICI monotherapy, dual immunotherapy was associated with a significantly higher risk of any grade of TRAEs (pooled RR = 1.04, 95% CI: 1.00–1.07, *p* = 0.03, *I* ^2 ^= 0%) (**Supplementary Figure 10A**).

In terms of grade 3–5 TRAES, no significant difference existed between the dual immunotherapy group and the other treatment groups (pooled RR = 1.08, 95% CI: 0.89–1.30, *p* = 0.44, *I* ^2 ^= 80%). As shown in the subgroup analysis, there was no significant difference between dual immunotherapy and chemotherapy (pooled RR = 0.85, 95% CI: 0.62–1.16, *p* = 0.31, *I* ^2 ^= 88%). In contrast, it was easily noticed that dual immunotherapy led to a high risk of G3–5 TRAEs when compared to ICI monotherapy (pooled RR = 1.29, 95% CI: 1.15–1.44, *p* < 0.0001, *I* ^2 ^= 0%) (**Supplementary Figure 10B**).

In addition, we investigated irAEs between dual immunotherapy and ICIs alone. Consistent with our suspicion, compared with ICIs alone, dual immunotherapy increased all grade irAEs (pooled RR = 1.48, 95% CI: 1.14–1.93, *p* = 0.003) and grade 3–5 irAEs (pooled RR = 4.90, 95% CI: 1.41–17.01, *p* = 0.01) (**Supplementary Figure S11**). Additional safety analyses are summarized in **Supplementary Tables 4, 5**.

### 3.3 Risk of bias assessment

The overall quality assessment of all involved randomized controlled trials was evaluated according to Cochrane Collaboration’s tool by Review Manager 5.4.1. Overall, nine studies included in this meta-analysis were high-quality RCTs with large information at low risk of bias (**Supplementary Figure S12**). Moreover, to make our combined outcomes robust, we conducted a sensitivity analysis by omitting specific studies. The pooled values did not change significantly in the condition that any one study was omitted (**Supplementary Figure 13**).

## 4 Discussion

Because of the complexity of immunoregulatory mechanisms and the heterogeneity of tumor and host, it is envisioned that combination immunotherapies will be required to efficiently treat a larger proportion of cancer patients (32). Our analysis illustrates that dual immunotherapy resulted in a significant improvement in OS (pooled HR = 0.76) and PFS (pooled HR = 0.75) among all tested PD-L1 expression levels compared to standard chemotherapy for the first-line treatment of advanced NSCLC. Compared with chemotherapy, the benefit of dual immunotherapy is predominantly seen in patients with PD-L1 expression of more than 50%, which was associated with a 30% reduction in the risk of death and a 37% reduction in the risk of disease progression. Importantly, dual immunotherapy also showed great advantages in patients with PD-L1 expression of less than 1%, which was associated with a 28% reduction in the risk of death and an 18% reduction in the risk of disease progression compared with chemotherapy. Although there is no statistical difference between dual immunotherapy and ICI monotherapy with regard to OS and ORR, dual immunotherapy is associated with a superior PFS benefit compared with single-agent ICIs (pooled HR = 0.75); these clinical benefits are most apparent in the subgroup with PD-L1 expression of less than 25% (pooled HR = 0.77) for whom anti-PD-1 monotherapy has been insufficient. These results were similar to those of another study that found that in the low PD-L1 expression condition, it was evident that the outcome of the combination therapy (anti-PD-1/L1 plus anti-CTLA-4 antibody) was superior to that of anti-PD-1 monotherapy when compared to the high PD-L1 expression group (29). These results indicate that using the expression level of PD-L1 as a predictive biomarker for dual immunotherapy remains has multiple challenges. However, owing to the majority of NSCLC patients having tumors with low, negative, or undetectable PD-L1 (33), our investigations have expanded the treatment options of most patients.

Given that the tumor mutational burden in lung and other cancers is also a predictive biomarker for checkpoint inhibition, we evaluated the clinical effectiveness of dual immunotherapy in different levels of TMB. As we expected, in terms of the high TMB subgroup, the outcome of dual immunotherapy led to significantly increased OS and PFS when compared with chemotherapy (HR = 0.69 for OS and HR = 0.71 for PFS) and a slight increase when compared to ICIs alone (HR = 0.85 for OS and HR = 0.76 for PFS); patients treated with dual immunotherapy also tended to have better ORR even though it did not reach statistical significance (pooled RR = 1.28). This finding warrants further investigation and prospective research of tumor mutational burden as a predictive biomarker for dual immunotherapy.

With regard to histologic type, compared to chemotherapy, a significant improvement in the OS and PFS in favor of dual immunotherapy has been observed also within the squamous group (HR = 0.64 for OS and HR = 0.66 for PFS) and the non-squamous group (HR = 0.75 for OS and HR = 0.78 for PFS), an observation that is contrary to the earlier belief that patients with squamous histologic type derive less benefit from checkpoint inhibitors (34). Because of the limited treatment strategies and poor prognosis of squamous non-small cell lung cancer, our observations may provide a novel treatment option for squamous NSCLC.

In addition, this meta-analysis further revealed that dual immunotherapy produced better OS benefits in patients with the following characteristics: younger age (<75 years old), male gender, ECOG PS = 0, smoker, no liver metastasis, with bone metastasis, with CNS metastasis, and using PD-1 antibodies. In terms of immuno-oncology (IO) drugs, a previous network meta-analysis reported a similar finding that anti-PD-1 therapy was superior to anti-PD-L1 therapy in terms of both PFS and tumor response (35). This may be due to the fact that PD-1 has been identified as the key immune checkpoint for regulating T- and B-cell response thresholds to antigens and exerts a pivotal role in regulating their cellular functions (12).

Because of limited data availability, we have only further compared objective response rates in patients who were treated with dual immunotherapy versus chemotherapy plus single immunotherapy in two clinical trials. The result demonstrates that there was no significant difference between dual immunotherapy and PD-1/L1 + chemotherapy (pooled RR = 0.88) with respect to the ORR value. In another indirect meta-analysis, patients with PD-L1 ≥ 50% advanced NSCLC who were treated with IO + CT combination had the best increase in ORR among three different IO-based treatment strategies (including combo IO), but the clinical outcome of ORR did not translate into a relevant improvement of patients’ survival (19). However, the long-term clinical efficacy between dual immunotherapy and chemotherapy plus single immunotherapy still required head-to-head randomized controlled trials.

Apart from the anti-PD-1/L1 combined with anti-CTLA-4 or TIGIT antibody that We discussed in this research, there is another combination therapy such as anti-PD-L1/TGF-β, which also enhances the effect of anti-PD-1/PD-L1 and relieve drug resistance (36). Previous studies demonstrate that therapeutic co-administration of TGF-β-blocking and anti-PD-L1 antibodies reduced TGF-β signaling in stromal cells, facilitated T-cell penetration into the center of tumors, and provoked vigorous anti-tumor immunity and tumor regression (37). Bintrafusp alfa (M7824) is a first-in-class bifunctional fusion protein simultaneously targeting TGF-β and PD-L1. In the early clinical studies, M7824 showed encouraging activity in advanced solid tumors, especially in NSCLC (38, 39). While progress for such approaches in clinical trials has been more difficult, M7824 has to be terminated in the later multiple phase II or III clinical trials because of poor efficacy (40). Recently, bispecific antibody (BsAb) targeting TGF-β and murine PD-L1 (termed YM101) also showed a superior anti-tumor effect when compared to the monotherapies in preclinical studies. Different from M7824, YM101 was developed based on the symmetric tetravalency BsAb technology (36). Furthermore, oral stimulation of interferon genes (STING) agonists such as manganese and MSA-2 synergized with YM101 could enhance naive T-cell activation, which improved the efficacy of YM101 in immune-excluded or immune-desert tumor models (41, 42). These data illustrate that the anti-TGF-β/PD-L1 combination strategy might provide a choice for cancer patients resistant to immune checkpoint inhibitors.

Nevertheless, the main concern of oncologists about combination therapy is the magnified risk of adverse events (43). Our meta-analysis demonstrated that there was no significant difference in any grade TRAEs or grade 3–5 TRAEs between patients who received dual immunotherapy and chemotherapy. In contrast, compared with ICI monotherapy, dual immunotherapy significantly increased the risk of developing any grade TRAEs and grade 3–5 TRAEs. Similarly, dual immunotherapy also produced a significant increase in immune-related adverse events as compared to ICI monotherapy. The frequency of treatment-attributed deaths was similar in both dual immunotherapy and ICI monotherapy.

However, this meta-analysis also has some limitations. First, the tissue PD-L1 assay is evaluated by immunohistochemistry (IHC) as determined by different FDA-approved assays, and the assessment of tumor mutational burden was based on both tumor tissue samples and blood samples, which may result in slightly biased detection results. Second, the interpretation of the results should be treated with caution since CheckMate 9LA experimental group contains two cycles of chemotherapy, and CITYSCAPE is the only clinical trial containing a monoclonal antibody to TIGIT, which evolves as a significant source of heterogeneity that show in subgroup analysis. Lastly, because of limited data, when we performed subgroup analysis according to PD-L1 expression levels, the number of included studies in each subgroup was small, which made subgroup analysis still unconvincing and merely suggestive. Notwithstanding these limitations, our meta-analysis, including the latest follow-up data from nine randomized clinical trials, has systematically assessed the clinical efficacy and safety of dual immunotherapy compared with either chemotherapy or ICI monotherapy.

## 5 Conclusions

We conclude that compared to chemotherapy, dual immunotherapy produced durable long-term clinical benefits in patients with advanced NSCLC with greater or less than 1% PD-L1 expression. In addition, the clinical efficacy of dual immunotherapy is more competitive in patients with high expression of TMB, squamous histology, and distant metastases. However, in the comparison of dual immunotherapy with ICI monotherapy, dual immunotherapy does not show obvious clinical benefits or improved safety of treatment. Thus, for patients with PD-L1 expression of more than 50%, we suggest that single-agent immunotherapy be continued, as it has the best efficacy and tolerability profile. Furthermore, due to the advantages in efficacy and safety of dual immunotherapy when compared with standard chemotherapy, dual immunotherapy is an optimized first-line choice for advanced NSCLC patients, particularly those with low or negative PD-L1 expression.

## Data availability statement

The original contributions presented in the study are included in the article/**Supplementary Material**. Further inquiries can be directed to the corresponding author.

## Author contributions

MA, XC, and YT contributed to the study conception and design. MT searched all related studies from databases, while MA and YT performed data extraction. JC and KT evaluated eligible study quality and potential bias risk. Statistical analysis was performed by MA. All authors contributed to the article and approved the submitted version.

## References

[1] Thai AA Solomon BJ Sequist LV Gainor JF Heist RS . Lung cancer. Lancet (London England) (2021) 398(10299):535–54. doi: 10.1016/s0140-6736(21)00312-3 34273294

[2] Travis WD Brambilla E Nicholson AG Yatabe Y Austin JHM Beasley MB . The 2015 world health organization classification of lung tumors: impact of genetic, clinical and radiologic advances since the 2004 classification. J Thorac Oncol (2015) 10(9):1243–60. doi: 10.1097/jto.0000000000000630 26291008

[3] Novello S Barlesi F Califano R Cufer T Ekman S Levra MG . Metastatic non-Small-Cell lung cancer: esmo clinical practice guidelines for diagnosis, treatment and follow-up. Ann Oncol (2016) 27(suppl 5):v1–v27. doi: 10.1093/annonc/mdw326 27664245

[4] Lu M Su Y . Immunotherapy in non-small cell lung cancer: the past, the present, and the future. Thorac Cancer (2019) 10(4):585–6. doi: 10.1111/1759-7714.13012 PMC6449275 30821103

[5] Miyazawa T Marushima H Saji H Kojima K Hoshikawa M Takagi M . Pd-L1 expression in non-Small-Cell lung cancer including various adenocarcinoma subtypes. Ann Thorac Cardiovasc Surg (2019) 25(1):1–9. doi: 10.5761/atcs.oa.18-00163 30282880 PMC6388302

[6] Herbst RS Giaccone G de Marinis F Reinmuth N Vergnenegre A Barrios CH . Atezolizumab for first-line treatment of pd-L1-Selected patients with nsclc. New Engl J Med (2020) 383(14):1328–39. doi: 10.1056/NEJMoa1917346 32997907

[7] Mok TSK Wu YL Kudaba I Kowalski DM Cho BC Turna HZ . Pembrolizumab versus chemotherapy for previously untreated, pd-L1-Expressing, locally advanced or metastatic non-Small-Cell lung cancer (Keynote-042): a randomised, open-label, controlled, phase 3 trial. Lancet (London England) (2019) 393(10183):1819–30. doi: 10.1016/s0140-6736(18)32409-7 30955977

[8] West H McCleod M Hussein M Morabito A Rittmeyer A Conter HJ . Atezolizumab in combination with carboplatin plus nab-paclitaxel chemotherapy compared with chemotherapy alone as first-line treatment for metastatic non-squamous non-Small-Cell lung cancer (Impower130): a multicentre, randomised, open-label, phase 3 trial. Lancet Oncol (2019) 20(7):924–37. doi: 10.1016/s1470-2045(19)30167-6 31122901

[9] Socinski MA Jotte RM Cappuzzo F Orlandi F Stroyakovskiy D Nogami N . Atezolizumab for first-line treatment of metastatic nonsquamous nsclc. New Engl J Med (2018) 378(24):2288–301. doi: 10.1056/NEJMoa1716948 29863955

[10] Brahmer J Reckamp KL Baas P Crinò L Eberhardt WE Poddubskaya E . Nivolumab versus docetaxel in advanced squamous-cell non-Small-Cell lung cancer. New Engl J Med (2015) 373(2):123–35. doi: 10.1056/NEJMoa1504627 PMC4681400 26028407

[11] Chen DS Mellman I . Oncology meets immunology: the cancer-immunity cycle. Immunity (2013) 39(1):1–10. doi: 10.1016/j.immuni.2013.07.012 23890059

[12] Wu M Huang Q Xie Y Wu X Ma H Zhang Y . Improvement of the anticancer efficacy of pd-1/Pd-L1 blockade *Via* combination therapy and pd-L1 regulation. J Hematol Oncol (2022) 15(1):24. doi: 10.1186/s13045-022-01242-2 35279217 PMC8917703

[13] Swart M Verbrugge I Beltman JB . Combination approaches with immune-checkpoint blockade in cancer therapy. Front Oncol (2016) 6:233. doi: 10.3389/fonc.2016.00233 27847783 PMC5088186

[14] Paz-Ares LG Ramalingam SS Ciuleanu TE Lee JS Urban L Caro RB . First-line nivolumab plus ipilimumab in advanced nsclc: 4-year outcomes from the randomized, open-label, phase 3 checkmate 227 part 1 trial. J Thorac Oncol Off Publ Int Assoc Study Lung Cancer (2022) 17(2):289–308. doi: 10.1016/j.jtho.2021.09.010 34648948

[15] Manieri NA Chiang EY Grogan JL . Tigit: a key inhibitor of the cancer immunity cycle. Trends Immunol (2017) 38(1):20–8. doi: 10.1016/j.it.2016.10.002 27793572

[16] Cho BC Abreu DR Hussein M Cobo M Patel AJ Secen N . Tiragolumab plus atezolizumab versus placebo plus atezolizumab as a first-line treatment for pd-L1-Selected non-Small-Cell lung cancer (Cityscape): primary and follow-up analyses of a randomised, double-blind, phase 2 study. Lancet Oncol (2022) 23(6):781–92. doi: 10.1016/s1470-2045(22)00226-1 35576957

[17] Boyer M Şendur MAN Rodríguez-Abreu D Park K Lee DH Çiçin I . Pembrolizumab plus ipilimumab or placebo for metastatic non-Small-Cell lung cancer with pd-L1 tumor proportion score ≥ 50%: randomized, double-blind phase iii keynote-598 study. J Clin Oncol Off J Am Soc Clin Oncol (2021) 39(21):2327–38. doi: 10.1200/jco.20.03579 33513313

[18] Higgins JP Altman DG Gøtzsche PC Jüni P Moher D Oxman AD . The cochrane collaboration's tool for assessing risk of bias in randomised trials. BMJ (Clinical Res ed) (2011) 343:d5928. doi: 10.1136/bmj.d5928 PMC3196245 22008217

[19] Passiglia F Galvano A Gristina V Barraco N Castiglia M Perez A . Is there any place for pd-1/Ctla-4 inhibitors combination in the first-line treatment of advanced nsclc?-a trial-level meta-analysis in pd-L1 selected subgroups. Trans Lung Cancer Res (2021) 10(7):3106–19. doi: 10.21037/tlcr-21-52 PMC8350096 34430351

[20] Eisenhauer EA Therasse P Bogaerts J Schwartz LH Sargent D Ford R . New response evaluation criteria in solid tumours: revised recist guideline (Version 1.1). Eur J Cancer (Oxford Engl 1990) (2009) 45(2):228–47. doi: 10.1016/j.ejca.2008.10.026 19097774

[21] Moher D Liberati A Tetzlaff J Altman DG . Preferred reporting items for systematic reviews and meta-analyses: the prisma statement. Int J Surg (2010) 8(5):336–41. doi: 10.1016/j.ijsu.2010.02.007 20171303

[22] Planchard D Reinmuth N Orlov S Fischer JR Sugawara S Mandziuk S . Arctic: Durvalumab with or without tremelimumab as third-line or later treatment of metastatic non-Small-Cell lung cancer. Ann Oncol Off J Eur Soc Med Oncol (2020) 31(5):609–18. doi: 10.1016/j.annonc.2020.02.006 32201234

[23] Rizvi NA Cho BC Reinmuth N Lee KH Luft A Ahn MJ . Durvalumab with or without tremelimumab vs standard chemotherapy in first-line treatment of metastatic non-small cell lung cancer: the mystic phase 3 randomized clinical trial. JAMA Oncol (2020) 6(5):661–74. doi: 10.1001/jamaoncol.2020.0237 PMC7146551 32271377

[24] Gettinger SN Redman MW Bazhenova L Hirsch FR Mack PC Schwartz LH . Nivolumab plus ipilimumab vs nivolumab for previously treated patients with stage iv squamous cell lung cancer: the lung-map S1400i phase 3 randomized clinical trial. JAMA Oncol (2021) 7(9):1368–77. doi: 10.1001/jamaoncol.2021.2209 PMC8283667 34264316

[25] Reck M Ciuleanu TE Cobo M Schenker M Zurawski B Menezes J . First-line nivolumab plus ipilimumab with two cycles of chemotherapy versus chemotherapy alone (Four cycles) in advanced non-Small-Cell lung cancer: checkmate 9la 2-year update. ESMO Open (2021) 6(5):100273. doi: 10.1016/j.esmoop.2021.100273 34607285 PMC8493593

[26] Johnson ML CB Luft A Alatorre-Alexander J . A Phase Iii Randomized, multi-center, open-label, comparative global study to determine the efficacy of durvalumab or durvalumab and tremelimumab in combination with platinum-based chemotherapy for first-line treatment in patients with metastatic non small-cell lung cancer (Nsclc) (Poseidon). (2017). doi: 10.1200/JCO.22.00975

[27] AstraZeneca . A phase iii randomized, open-label, multi-center, global study of Medi4736 in combination with tremelimumab therapy versus standard of care platinum-based chemotherapy in first line treatment of patients with advanced or metastatic non small-cell lung cancer (Nsclc) (Neptune): AstraZeneca (2015). Available at: https://clinicaltrials.gov/ct2/show/record/NCT02542293.

[28] Rodriguez Abreu D Reck M Şendur N Park K Lee DH Cicin I . 6mo pembrolizumab plus ipilimumab or placebo in previously untreated metastatic nsclc with pd-L1 tumor proportion score ≥50%: keynote-598 3-year follow-up. Ann Oncol (2022) 33:S30–S1. doi: 10.1016/j.annonc.2022.02.015

[29] Wu K Yi M Qin S Chu Q Zheng X Wu K . The efficacy and safety of combination of pd-1 and ctla-4 inhibitors: a meta-analysis. Exp Hematol Oncol (2019) 8:26. doi: 10.1186/s40164-019-0150-0 31673481 PMC6815037

[30] Yarchoan M Hopkins A Jaffee EM . Tumor mutational burden and response rate to pd-1 inhibition. New Engl J Med (2017) 377(25):2500–1. doi: 10.1056/NEJMc1713444 PMC6549688 29262275

[31] Hellmann MD Ciuleanu TE Pluzanski A Lee JS Otterson GA Audigier-Valette C . Nivolumab plus ipilimumab in lung cancer with a high tumor mutational burden. New Engl J Med (2018) 378(22):2093–104. doi: 10.1056/NEJMoa1801946 PMC7193684 29658845

[32] Zou W Wolchok JD Chen L . Pd-L1 (B7-H1) and pd-1 pathway blockade for cancer therapy: mechanisms, response biomarkers, and combinations. Sci Trans Med (2016) 8(328):328rv4. doi: 10.1126/scitranslmed.aad7118 PMC4859220 26936508

[33] Rimm DL Han G Taube JM Yi ES Bridge JA Flieder DB . A prospective, multi-institutional, pathologist-based assessment of 4 immunohistochemistry assays for pd-L1 expression in non-small cell lung cancer. JAMA Oncol (2017) 3(8):1051–8. doi: 10.1001/jamaoncol.2017.0013 PMC5650234 28278348

[34] Gettinger S Rizvi NA Chow LQ Borghaei H Brahmer J Ready N . Nivolumab monotherapy for first-line treatment of advanced non-Small-Cell lung cancer. J Clin Oncol Off J Am Soc Clin Oncol (2016) 34(25):2980–7. doi: 10.1200/jco.2016.66.9929 PMC5569692 27354485

[35] You W Liu M Miao JD Liao YQ Song YB Cai DK . A network meta-analysis comparing the efficacy and safety of anti-Pd-1 with anti-Pd-L1 in non-small cell lung cancer. J Cancer (2018) 9(7):1200–6. doi: 10.7150/jca.22361 PMC5907668 29675101

[36] Yi M Zhang J Li A Niu M Yan Y Jiao Y . The construction, expression, and enhanced anti-tumor activity of Ym101: a bispecific antibody simultaneously targeting tgf-Β and pd-L1. J Hematol Oncol (2021) 14(1):27. doi: 10.1186/s13045-021-01045-x 33593403 PMC7885589

[37] Mariathasan S Turley SJ Nickles D Castiglioni A Yuen K Wang Y . Tgfβ attenuates tumour response to pd-L1 blockade by contributing to exclusion of T cells. Nature (2018) 554(7693):544–8. doi: 10.1038/nature25501 PMC6028240 29443960

[38] Paz-Ares L Kim TM Vicente D Felip E Lee DH Lee KH . Bintrafusp Alfa, a bifunctional fusion protein targeting tgf-Β and pd-L1, in second-line treatment of patients with nsclc: results from an expansion cohort of a phase 1 trial. J Thorac Oncol Off Publ Int Assoc Study Lung Cancer (2020) 15(7):1210–22. doi: 10.1016/j.jtho.2020.03.003 PMC8210474 32173464

[39] Strauss J Heery CR Schlom J Madan RA Cao L Kang Z . Phase I trial of M7824 (Msb0011359c), a bifunctional fusion protein targeting pd-L1 and tgfβ, in advanced solid tumors. Clin Cancer Res an Off J Am Assoc Cancer Res (2018) 24(6):1287–95. doi: 10.1158/1078-0432.Ccr-17-2653 PMC7985967 29298798

[40] Metropulos AE Munshi HG Principe DR . The difficulty in translating the preclinical success of combined tgfβ and immune checkpoint inhibition to clinical trial. EBioMedicine (2022) 86:104380. doi: 10.1016/j.ebiom.2022.104380 36455409 PMC9706619

[41] Yi M Niu M Zhang J Li S Zhu S Yan Y . Combine and conquer: manganese synergizing anti-Tgf-Β/Pd-L1 bispecific antibody Ym101 to overcome immunotherapy resistance in non-inflamed cancers. J Hematol Oncol (2021) 14(1):146. doi: 10.1186/s13045-021-01155-6 34526097 PMC8442312

[42] Yi M Niu M Wu Y Ge H Jiao D Zhu S . Combination of oral sting agonist msa-2 and anti-Tgf-Β/Pd-L1 bispecific antibody Ym101: a novel immune cocktail therapy for non-inflamed tumors. J Hematol Oncol (2022) 15(1):142. doi: 10.1186/s13045-022-01363-8 36209176 PMC9548169

[43] Seidel JA Otsuka A Kabashima K . Anti-Pd-1 and anti-Ctla-4 therapies in cancer: mechanisms of action, efficacy, and limitations. Front Oncol (2018) 8:86. doi: 10.3389/fonc.2018.00086 29644214 PMC5883082

