# Supplementary material for: Checkpoint inhibitors as dual immunotherapy in advanced non-small cell lung cancer: a meta-analysis
Source: Front Oncol. 2023 Jun 15;13:1146905. doi: 10.3389/fonc.2023.1146905 (PMC10311062; doi:10.3389/fonc.2023.1146905)
Supplement: Supplementary file 1 [file DataSheet_1.zip › Supplementary Figure 1.pdf]

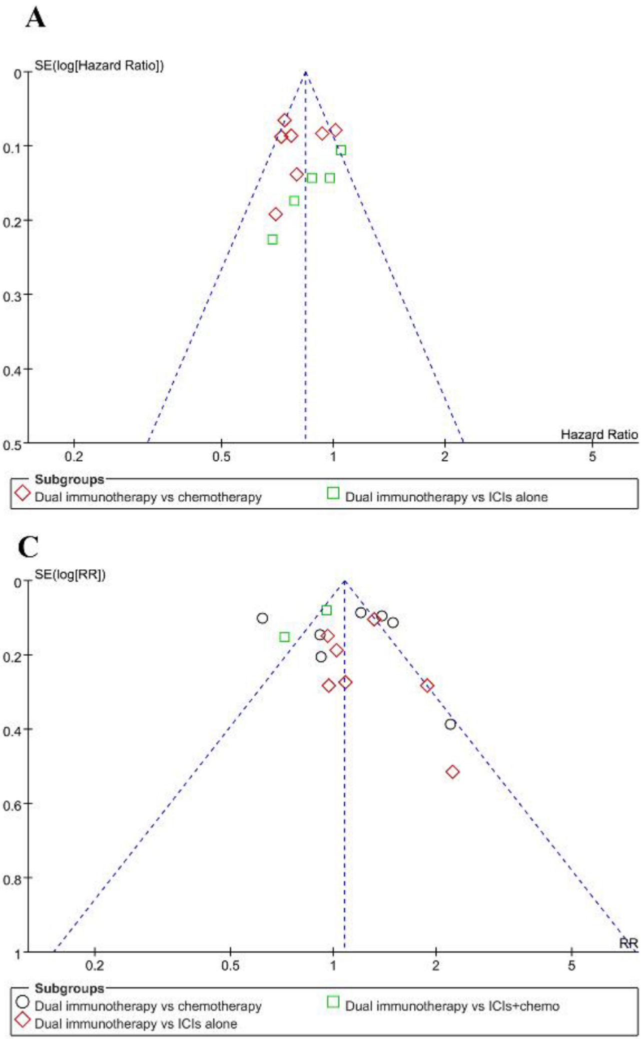


**Supplementary Figure 1.** Funnel plot comparing Hazard Ratios for overall survival(A) and progression-free survival(B), and Risk Ratios for Objective Response Rate(C).
