# Supplementary material for: Checkpoint inhibitors as dual immunotherapy in advanced non-small cell lung cancer: a meta-analysis
Source: Front Oncol. 2023 Jun 15;13:1146905. doi: 10.3389/fonc.2023.1146905 (PMC10311062; doi:10.3389/fonc.2023.1146905)
Supplement: Supplementary file 1 [file DataSheet_1.zip › Supplementary Figure 10.pdf]

A

Study or Subgroup	Dual immunotherapy Events	Dual immunotherapy Total	Control Events	Control Total	Weight	Risk Ratio M-H, Random, 95% CI
**1.5.1 Dual immunotherapy vs chemo**						
ARCTIC, 2020	108	173	89	110	8.4%	0.77 [0.67, 0.89]
Checkmate 227part1, 2022	442	576	469	570	11.4%	0.93 [0.88, 0.99]
Checkmate 9LA, 2021	328	358	306	349	11.6%	1.04 [0.99, 1.10]
MYSTIC, 2020	223	371	292	352	10.2%	0.72 [0.66, 0.80]
NEPTUNE, 2022	328	410	329	413	11.1%	1.00 [0.94, 1.08]
NEPTUNE, 2022	50	78	62	82	6.4%	0.85 [0.69, 1.04]
**Subtotal (95% CI)**		**1966**		**1876**	**59.0%**	**0.89 [0.79, 1.00]**
Total events	1479		1547			
Heterogeneity: Tau² = 0.02; Chi² = 62.64, df = 5 (P < 0.00001); I² = 92%						
Test for overall effect: Z = 1.96 (P = 0.05)						
**1.5.2 Dual immunotherapy VS ICIs alone**						
ARCTIC, 2020	108	173	73	117	7.2%	1.00 [0.83, 1.20]
ARCTIC, 2020	108	173	38	60	5.9%	0.99 [0.79, 1.23]
CITYSCAPE, 2022	55	67	48	68	6.9%	1.16 [0.96, 1.41]
Keynote 598, 2022	272	282	263	281	11.8%	1.03 [0.99, 1.07]
MYSTIC, 2020	223	371	200	369	9.2%	1.11 [0.98, 1.26]
**Subtotal (95% CI)**		**1066**		**895**	**41.0%**	**1.04 [1.00, 1.07]**
Total events	766		622			
Heterogeneity: Tau² = 0.00; Chi² = 3.90, df = 4 (P = 0.42); I² = 0%						
Test for overall effect: Z = 2.13 (P = 0.03)						
**Total (95% CI)**		**3032**		**2771**	**100.0%**	**0.96 [0.89, 1.03]**
Total events	2245		2169			
Heterogeneity: Tau² = 0.01; Chi² = 80.92, df = 10 (P < 0.00001); I² = 88%						
Test for overall effect: Z = 1.16 (P = 0.25)						
Test for subgroup differences: Chi² = 6.13, df = 1 (P = 0.01), I² = 83.7%						

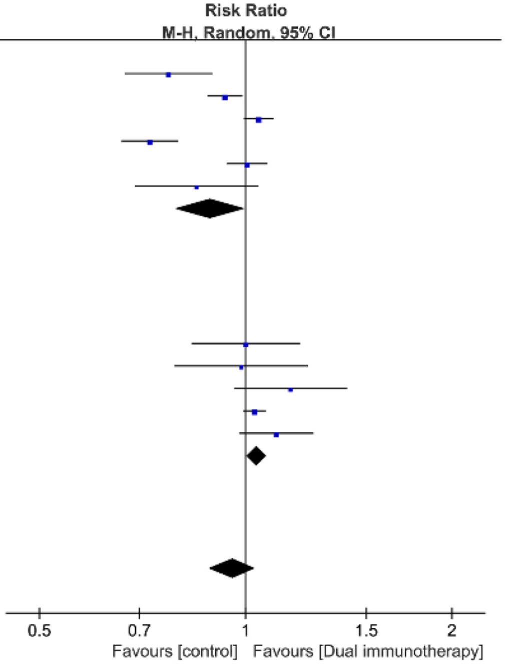

B

Study or Subgroup	Dual immunotherapy Events	Dual immunotherapy Total	Control Events	Control Total	Weight	Risk Ratio M-H, Random, 95% CI
**1.6.1 Dual immunotherapy vs chemo**						
ARCTIC, 2020	38	173	40	110	8.9%	0.60 [0.42, 0.88]
Checkmate 227part1, 2022	189	576	205	570	12.7%	0.91 [0.78, 1.07]
Checkmate 9LA, 2021	173	358	132	349	12.5%	1.28 [1.08, 1.52]
MYSTIC, 2020	85	371	119	352	11.4%	0.68 [0.53, 0.86]
**Subtotal (95% CI)**		**1478**		**1381**	**45.5%**	**0.85 [0.62, 1.16]**
Total events	485		496			
Heterogeneity: Tau² = 0.09; Chi² = 25.22, df = 3 (P < 0.0001); I² = 88%						
Test for overall effect: Z = 1.01 (P = 0.31)						
**1.6.2 Dual immunotherapy vs PD-L1/1**						
ARCTIC, 2020	38	173	15	117	6.3%	1.71 [0.99, 2.97]
ARCTIC, 2020	38	173	14	60	6.5%	0.94 [0.55, 1.61]
CITYSCAPE, 2022	35	67	27	68	9.0%	1.32 [0.91, 1.91]
Keynote 598, 2022	176	282	140	281	12.8%	1.25 [1.08, 1.45]
MYSTIC, 2020	85	371	55	369	10.1%	1.54 [1.13, 2.09]
The Lung-MAP S1400I, 2021	49	124	41	123	9.7%	1.19 [0.85, 1.65]
**Subtotal (95% CI)**		**1190**		**1018**	**54.5%**	**1.29 [1.15, 1.44]**
Total events	421		292			
Heterogeneity: Tau² = 0.00; Chi² = 4.08, df = 5 (P = 0.54); I² = 0%						
Test for overall effect: Z = 4.39 (P < 0.0001)						
**Total (95% CI)**		**2668**		**2399**	**100.0%**	**1.08 [0.89, 1.30]**
Total events	906		788			
Heterogeneity: Tau² = 0.06; Chi² = 45.48, df = 9 (P < 0.00001); I² = 80%						
Test for overall effect: Z = 0.77 (P = 0.44)						
Test for subgroup differences: Chi² = 5.92, df = 1 (P = 0.02), I² = 83.1%						

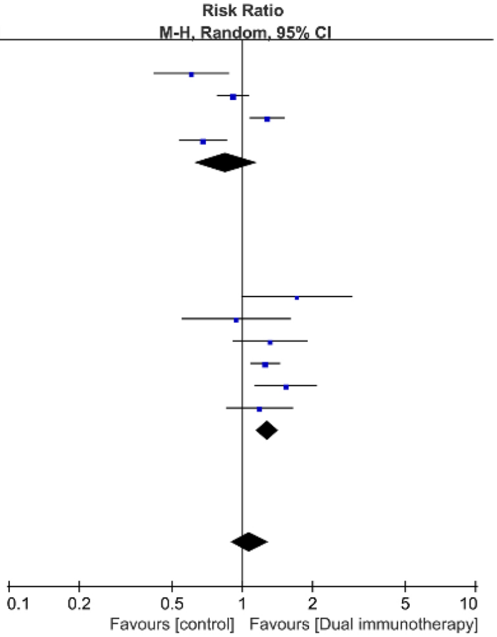

Supplementary Figure 10. Forest plot of Risk ratio (RR). Comparison of any grade treatment-related adverse events (TRAEs) (A) and grade 3-5 adverse events (Grade3-5AEs) (B) between dual immunotherapy and other treatments. ICIs: immune checkpoint inhibitors.
