# Supplementary material for: Checkpoint inhibitors as dual immunotherapy in advanced non-small cell lung cancer: a meta-analysis
Source: Front Oncol. 2023 Jun 15;13:1146905. doi: 10.3389/fonc.2023.1146905 (PMC10311062; doi:10.3389/fonc.2023.1146905)
Supplement: Supplementary file 1 [file DataSheet_1.zip › Supplementary Figure 11.pdf]

Study or Subgroup	dual immunotherapy Events	dual immunotherapy Total	ICIs alone Events	ICIs alone Total	Weight	Risk Ratio M-H, Random, 95% CI
**1.10.1 all IMAEs**						
CITYSCAPE, 2022	51	67	32	68	18.4%	1.62 [1.22, 2.15]
Keynote 598, 2022	126	282	91	281	19.3%	1.38 [1.11, 1.71]
MYSTIC, 2020	105	371	50	369	18.1%	2.09 [1.54, 2.83]
The Lung-MAP S1400I, 2021	82	124	73	123	19.5%	1.11 [0.92, 1.35]
**Subtotal (95% CI)**		**844**		**841**	**75.3%**	**1.48 [1.14, 1.93]**
Total events	364		246			
Heterogeneity: Tau² = 0.06; Chi² = 13.92, df = 3 (P = 0.003); I² = 78%						
Test for overall effect: Z = 2.94 (P = 0.003)						
**1.10.2 G3-5 IMAEs**						
Keynote 598, 2022	57	282	6	281	10.4%	9.47 [4.15, 21.60]
MYSTIC, 2020	44	371	16	369	14.3%	2.74 [1.57, 4.76]
**Subtotal (95% CI)**		**653**		**650**	**24.7%**	**4.90 [1.41, 17.01]**
Total events	101		22			
Heterogeneity: Tau² = 0.68; Chi² = 6.30, df = 1 (P = 0.01); I² = 84%						
Test for overall effect: Z = 2.50 (P = 0.01)						
**Total (95% CI)**		**1497**		**1491**	**100.0%**	**1.98 [1.36, 2.89]**
Total events	465		268			
Heterogeneity: Tau² = 0.18; Chi² = 44.91, df = 5 (P < 0.00001); I² = 89%						
Test for overall effect: Z = 3.54 (P = 0.0004)						
Test for subgroup differences: Chi² = 3.40, df = 1 (P = 0.07), I² = 70.6%						

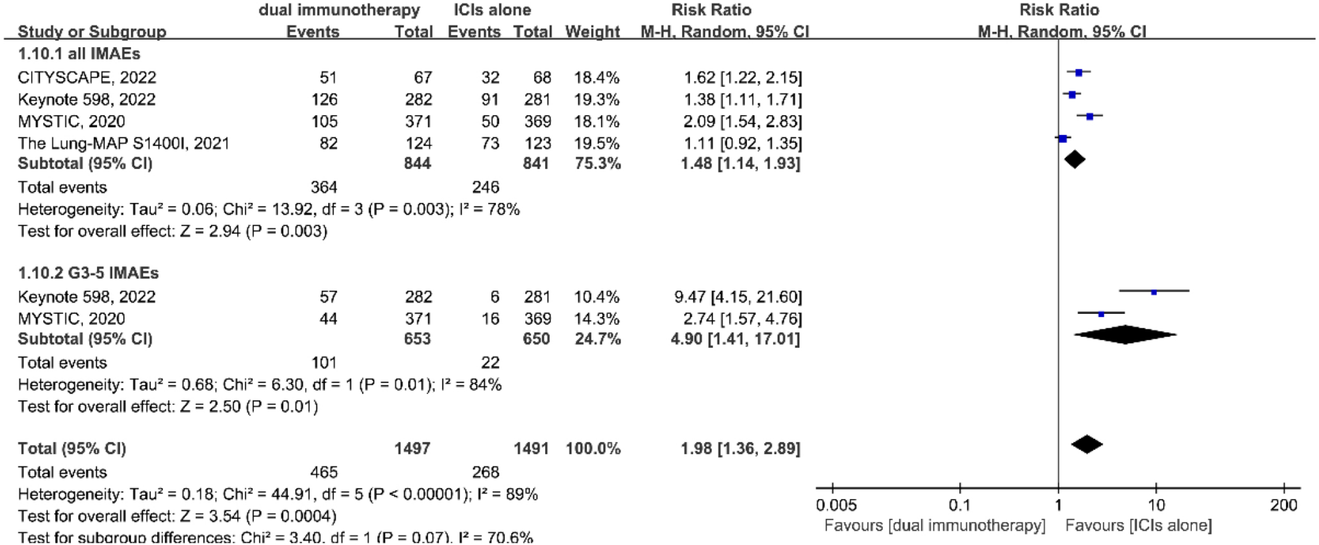

Supplementary Figure 11. Forest plot of Risk ratio (RR). Comparison of Immune-related adverse rates (irAEs) and grade 3-5 IMAEs (G3-5 IMAEs) between dual immunotherapy and ICIs alone. ICIs: immune checkpoint inhibitors.
