# Supplementary material for: Checkpoint inhibitors as dual immunotherapy in advanced non-small cell lung cancer: a meta-analysis
Source: Front Oncol. 2023 Jun 15;13:1146905. doi: 10.3389/fonc.2023.1146905 (PMC10311062; doi:10.3389/fonc.2023.1146905)
Supplement: Supplementary file 1 [file DataSheet_1.zip › Supplementary Figure 13.pdf]

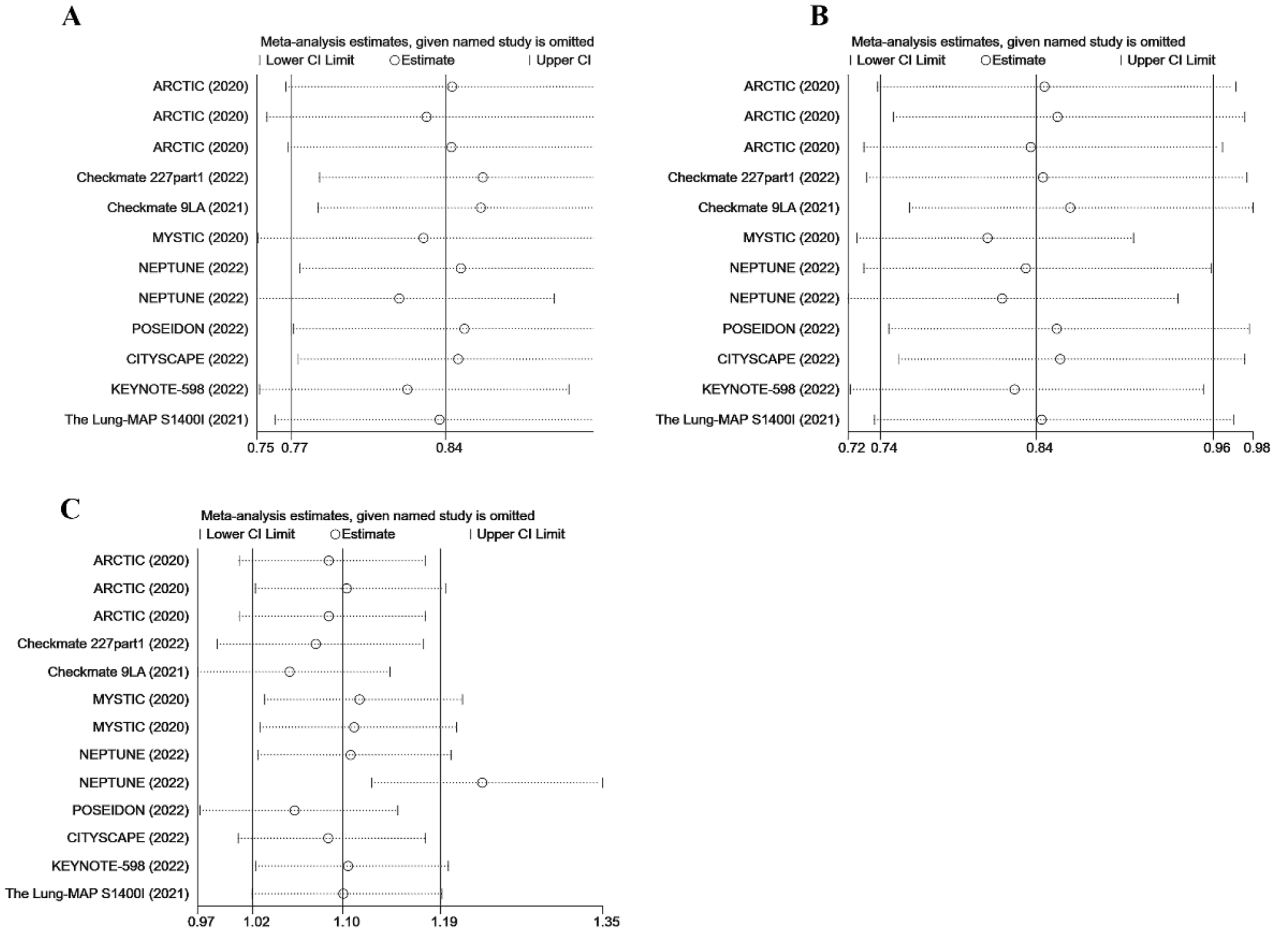

Supplementary Figure 13. Sensitivity analysis of Overall survival (OS)(A), Progression-free survival (PFS) (B), objective response rate (ORR) (C) by repeating the pooled analysis with one study omitted at a time.
