# Supplementary material for: Checkpoint inhibitors as dual immunotherapy in advanced non-small cell lung cancer: a meta-analysis
Source: Front Oncol. 2023 Jun 15;13:1146905. doi: 10.3389/fonc.2023.1146905 (PMC10311062; doi:10.3389/fonc.2023.1146905)
Supplement: Supplementary file 1 [file DataSheet_1.zip › Supplementary Figure 2.pdf]

PRISMA 2020 flow diagram for new systematic reviews which included searches of databases and registers only

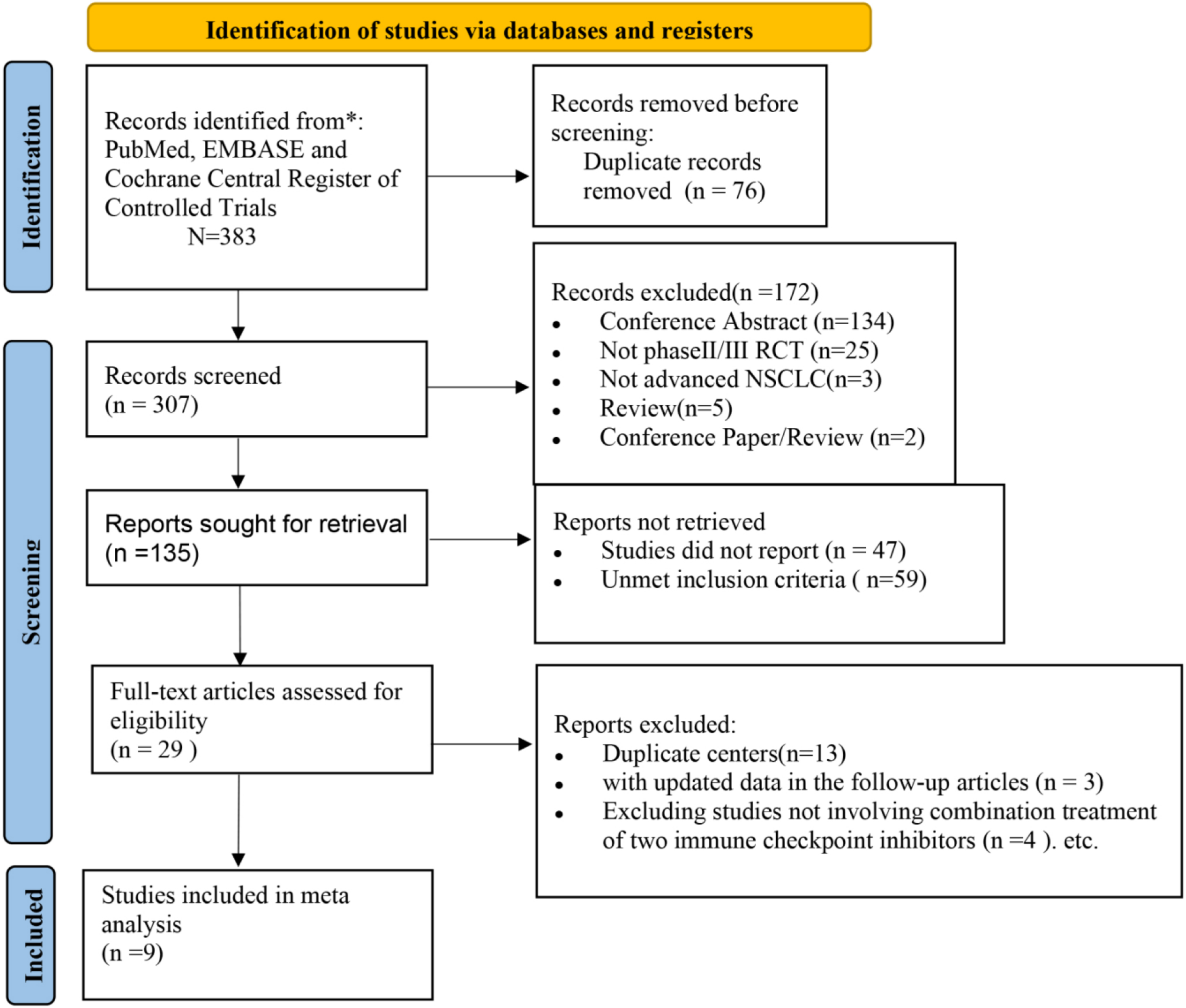

Supplementary Figure 2. PRISMA 2020 flow diagram showing selection process for the trials included in the meta-analysis.
