# Supplementary material for: Checkpoint inhibitors as dual immunotherapy in advanced non-small cell lung cancer: a meta-analysis
Source: Front Oncol. 2023 Jun 15;13:1146905. doi: 10.3389/fonc.2023.1146905 (PMC10311062; doi:10.3389/fonc.2023.1146905)
Supplement: Supplementary file 1 [file DataSheet_1.zip › Supplementary Figure 3.pdf]

Study or Subgroup	Dual immunotherapy Events	Dual immunotherapy Total	Control Events	Control Total	Weight	Risk Ratio M-H, Fixed, 95% CI
**4.3.1 Dual immunotherapy vs chemotherapy**						
ARCTIC, 2020	26	174	8	118	0.8%	2.20 [1.03, 4.70]
Checkmate 227part1, 2022	195	583	162	583	14.2%	1.20 [1.01, 1.43]
Checkmate 9LA, 2021	137	361	91	358	8.0%	1.49 [1.20, 1.86]
MYSTIC, 2020	56	163	61	162	5.4%	0.91 [0.68, 1.22]
NEPTUNE, 2022	28	78	32	82	2.7%	0.92 [0.62, 1.37]
NEPTUNE, 2022	106	410	172	412	15.0%	0.62 [0.51, 0.76]
POSEIDON, 2022	154	335	110	332	9.7%	1.39 [1.15, 1.68]
**Subtotal (95% CI)**		**2104**		**2047**	**55.8%**	**1.09 [1.00, 1.19]**
Total events	702		636			
Heterogeneity: $Chi^2 = 51.34$, df = 6 ($P < 0.00001$); $I^2 = 88\%$						
Test for overall effect: $Z = 1.97$ ($P = 0.05$)						
**4.3.2 Dual immunotherapy vs ICIs alone**						
ARCTIC, 2020	26	174	4	60	0.5%	2.24 [0.82, 6.16]
ARCTIC, 2020	26	174	18	117	1.9%	0.97 [0.56, 1.69]
Checkmate 227part1, 2022	144	396	109	396	9.5%	1.32 [1.07, 1.62]
CITYSCAPE, 2022	26	67	14	68	1.2%	1.88 [1.08, 3.28]
Keynote 598, 2022	47	284	46	284	4.0%	1.02 [0.70, 1.48]
MYSTIC, 2020	56	163	58	163	5.1%	0.97 [0.72, 1.30]
The Lung-MAP S1400I, 2021	23	124	21	123	1.8%	1.09 [0.64, 1.86]
**Subtotal (95% CI)**		**1382**		**1211**	**24.1%**	**1.20 [1.05, 1.38]**
Total events	348		270			
Heterogeneity: $Chi^2 = 8.32$, df = 6 ($P = 0.22$); $I^2 = 28\%$						
Test for overall effect: $Z = 2.59$ ($P = 0.010$)						
**4.3.3 Dual immunotherapy vs ICIs+chemo**						
Checkmate 227part1, 2022	51	187	67	177	6.0%	0.72 [0.53, 0.97]
POSEIDON, 2022	155	335	160	330	14.1%	0.95 [0.81, 1.12]
**Subtotal (95% CI)**		**522**		**507**	**20.1%**	**0.88 [0.77, 1.02]**
Total events	206		227			
Heterogeneity: $Chi^2 = 2.65$, df = 1 ($P = 0.10$); $I^2 = 62\%$						
Test for overall effect: $Z = 1.69$ ($P = 0.09$)						
**Total (95% CI)**		**4008**		**3765**	**100.0%**	**1.08 [1.01, 1.15]**
Total events	1256		1133			
Heterogeneity: $Chi^2 = 70.90$, df = 15 ($P < 0.00001$); $I^2 = 79\%$						
Test for overall effect: $Z = 2.19$ ($P = 0.03$)						
Test for subgroup differences: $Chi^2 = 9.76$, df = 2 ($P = 0.008$), $I^2 = 79.5\%$						

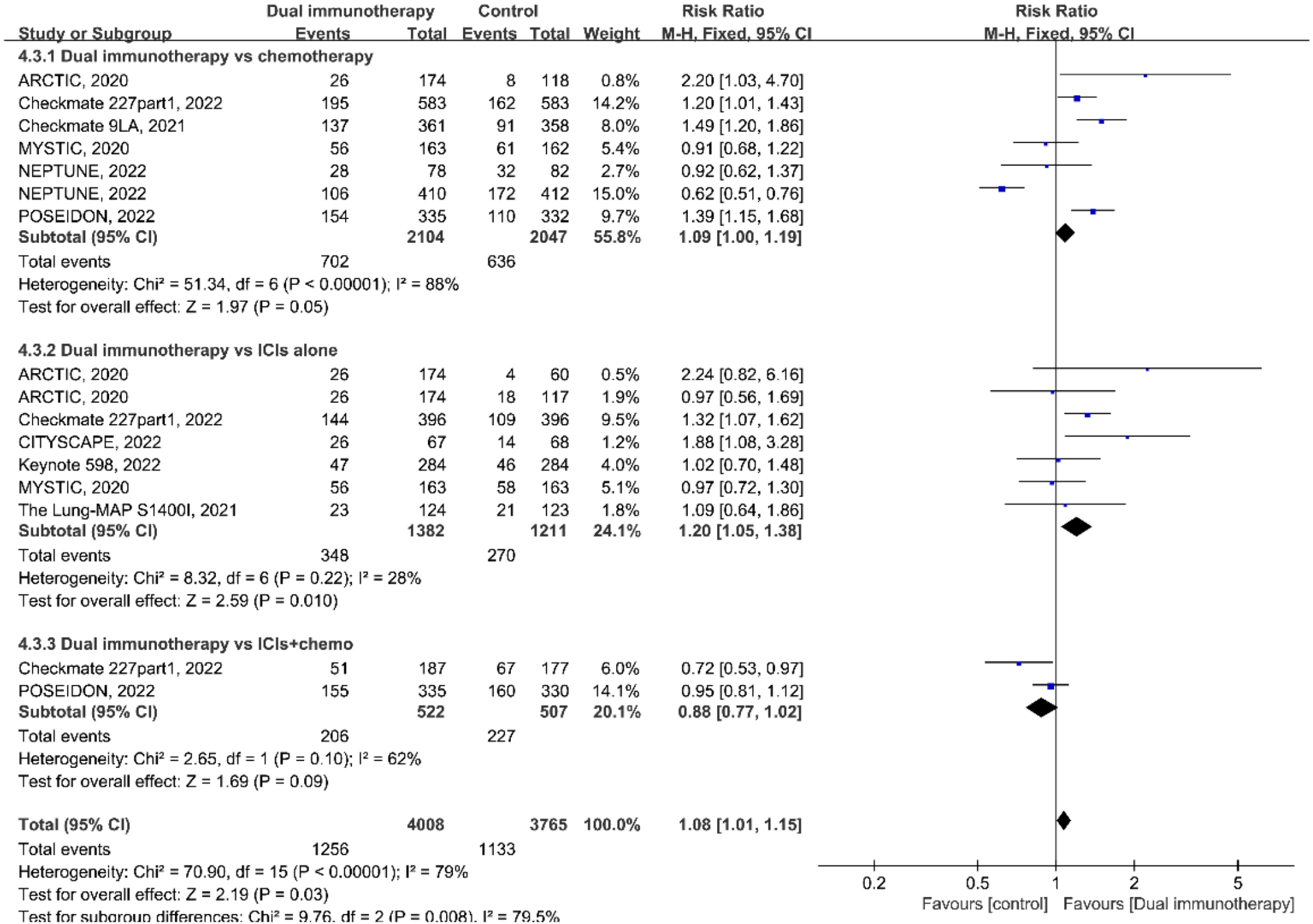

Supplementary Figure 3. Forest plot of Risk ratio (RR). Comparison of objective response rates (ORR) between dual immunotherapy and other treatments. ICIs: immune checkpoint inhibitors.
