# Supplementary material for: Checkpoint inhibitors as dual immunotherapy in advanced non-small cell lung cancer: a meta-analysis
Source: Front Oncol. 2023 Jun 15;13:1146905. doi: 10.3389/fonc.2023.1146905 (PMC10311062; doi:10.3389/fonc.2023.1146905)
Supplement: Supplementary file 1 [file DataSheet_1.zip › Supplementary Figure 4.pdf]

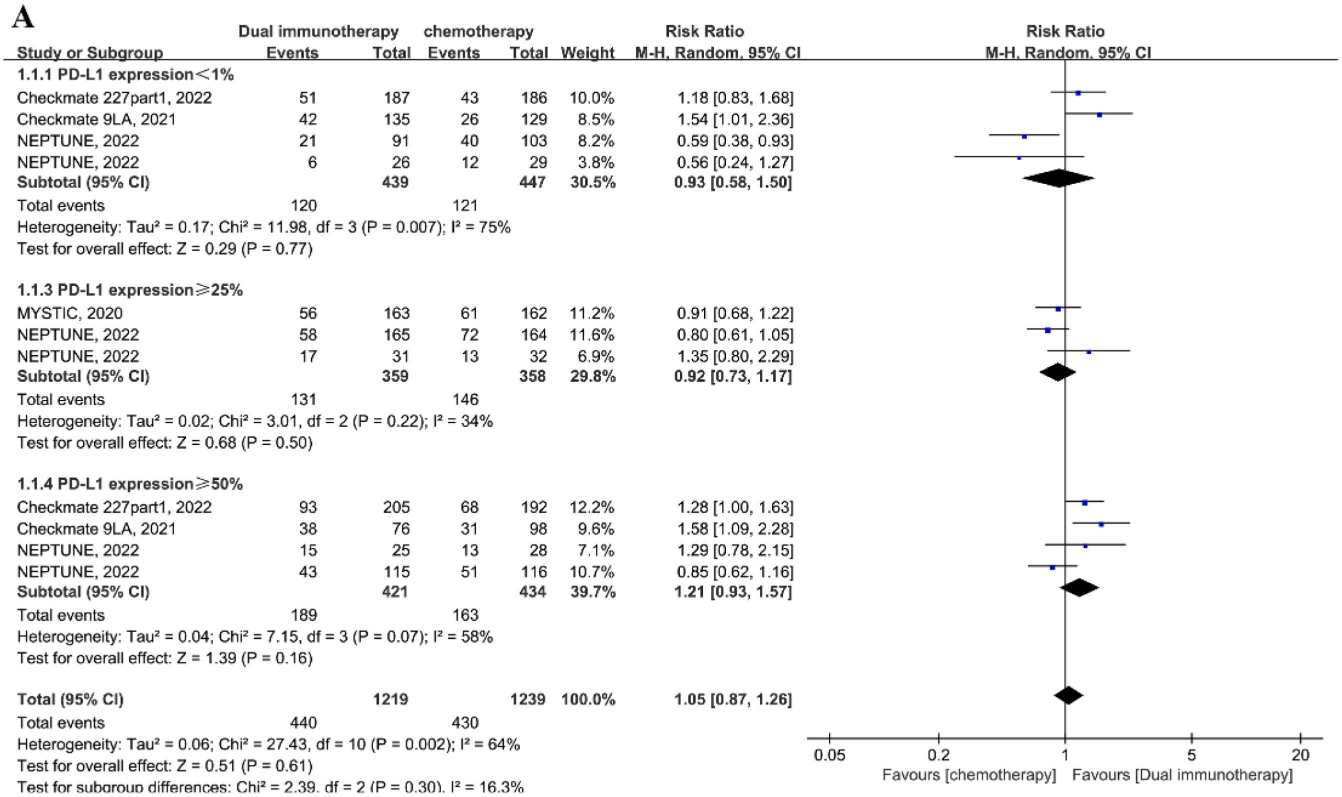

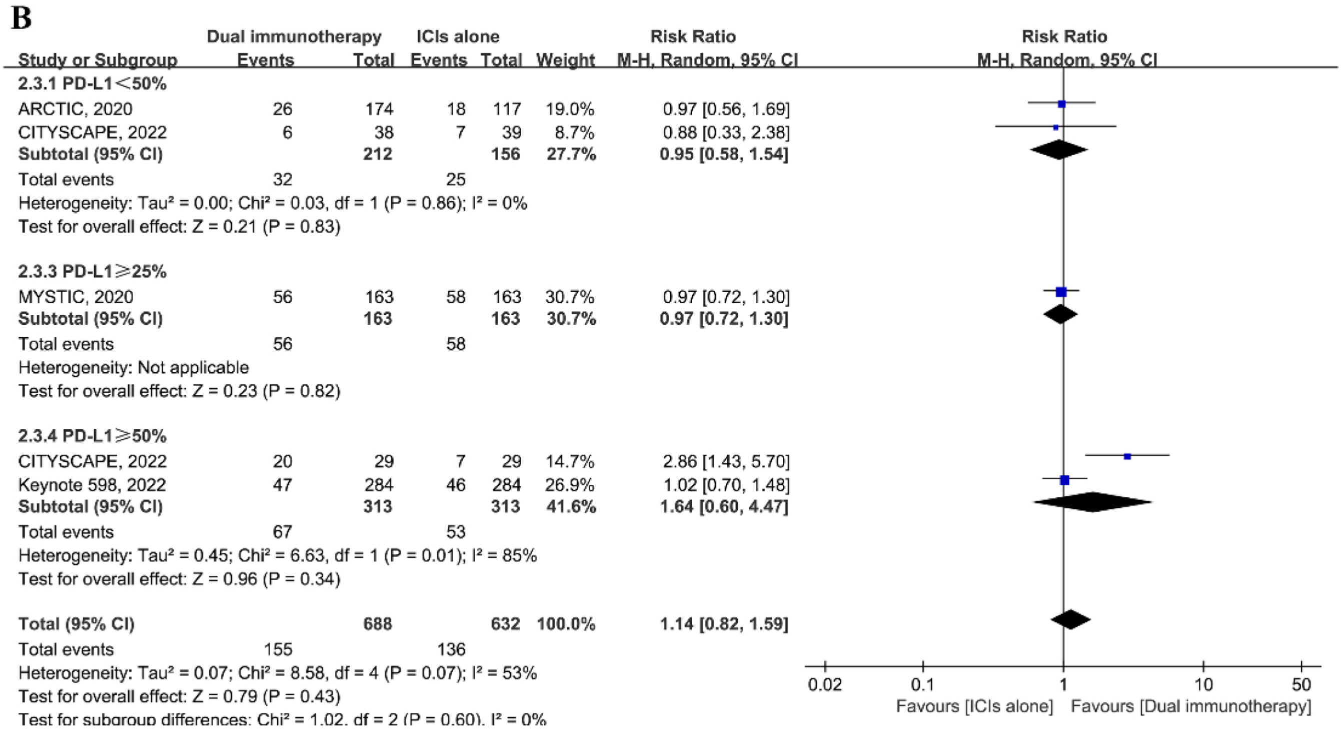

Supplementary Figure 4. Forest plot of Risk ratio (RR). Subgroup analysis of objective response rates (ORR) between dual immunotherapy and either chemotherapy(A) or ICIs monotherapy(B) depending on PD-L1 expression. ICIs: immune checkpoint inhibitors.
