# Supplementary material for: Checkpoint inhibitors as dual immunotherapy in advanced non-small cell lung cancer: a meta-analysis
Source: Front Oncol. 2023 Jun 15;13:1146905. doi: 10.3389/fonc.2023.1146905 (PMC10311062; doi:10.3389/fonc.2023.1146905)
Supplement: Supplementary file 1 [file DataSheet_1.zip › Supplementary Figure 6.pdf]

Study or Subgroup	Dual immunotherapy Events	Dual immunotherapy Total	Control Events	Control Total	Weight	Risk Ratio M-H, Random, 95% CI
**4.11.1 Dual immunotherapy vs chemotherapy**						
Checkmate 227part1, 2022	63	139	43	160	22.1%	1.69 [1.23, 2.31]
MYSTIC, 2020	31	64	15	70	18.2%	2.26 [1.35, 3.78]
NEPTUNE, 2022	19	69	26	60	18.9%	0.64 [0.39, 1.03]
NEPTUNE, 2022	29	77	34	80	20.8%	0.89 [0.60, 1.30]
**Subtotal (95% CI)**		**349**		**370**	**80.0%**	**1.21 [0.72, 2.05]**
Total events	142		118			
Heterogeneity: Tau² = 0.24; Chi² = 19.33, df = 3 (P = 0.0002); I² = 84%						
Test for overall effect: Z = 0.71 (P = 0.48)						
**4.11.2 Dual immunotherapy vs ICIs alone**						
MYSTIC, 2020	31	64	23	77	20.0%	1.62 [1.06, 2.48]
**Subtotal (95% CI)**		**64**		**77**	**20.0%**	**1.62 [1.06, 2.48]**
Total events	31		23			
Heterogeneity: Not applicable						
Test for overall effect: Z = 2.23 (P = 0.03)						
**Total (95% CI)**		**413**		**447**	**100.0%**	**1.28 [0.84, 1.95]**
Total events	173		141			
Heterogeneity: Tau² = 0.18; Chi² = 20.49, df = 4 (P = 0.0004); I² = 80%						
Test for overall effect: Z = 1.17 (P = 0.24)						
Test for subgroup differences: Chi² = 0.72, df = 1 (P = 0.40), I² = 0%						

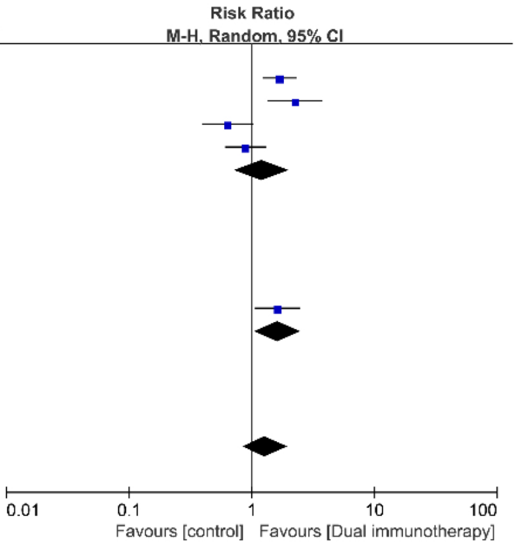

Supplementary Figure 6. Forest plot of risk ratio (RR). Comparison of Objective response rates (ORR) between dual immunotherapy and other treatments in high TMB subgroup. TMB: tumor mutational burden.
