# Supplementary material for: Checkpoint inhibitors as dual immunotherapy in advanced non-small cell lung cancer: a meta-analysis
Source: Front Oncol. 2023 Jun 15;13:1146905. doi: 10.3389/fonc.2023.1146905 (PMC10311062; doi:10.3389/fonc.2023.1146905)
Supplement: Supplementary file 1 [file DataSheet_1.zip › Supplementary Figure 8.pdf]

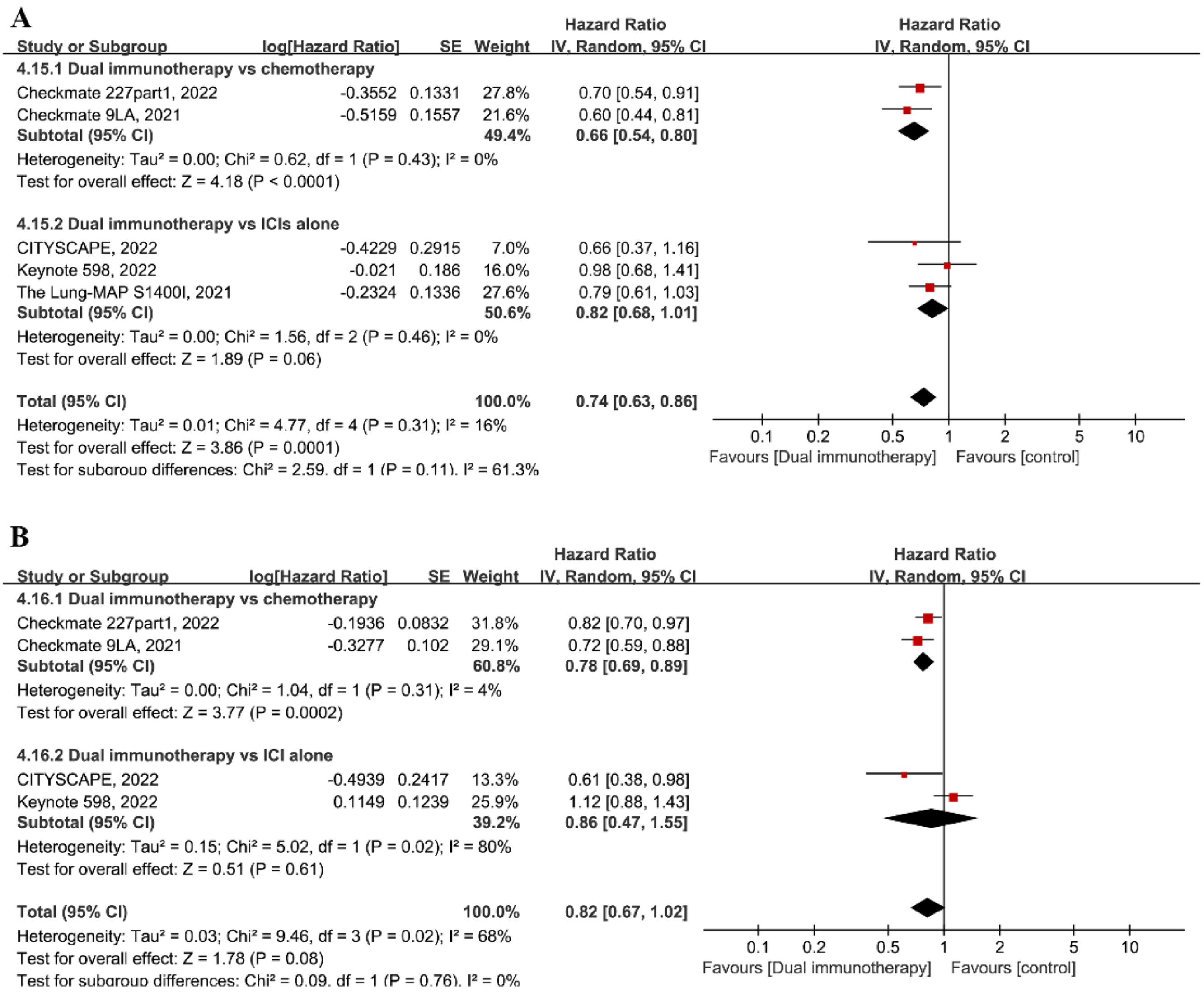

Supplementary Figure 8. Forest plot of hazard ratio (HR). Comparison of Progression-free survival (PFS) between dual immunotherapy and other treatments in squamous subgroup(A); in non-squamous subgroup (B). ICIs: immune checkpoint inhibitors.
