# Supplementary material for: Checkpoint inhibitors as dual immunotherapy in advanced non-small cell lung cancer: a meta-analysis
Source: Front Oncol. 2023 Jun 15;13:1146905. doi: 10.3389/fonc.2023.1146905 (PMC10311062; doi:10.3389/fonc.2023.1146905)
Supplement: Supplementary file 2 [file Table_1.docx]

**Supplementary Table 1. Stratification of clinical outcomes measures considered in this pooled analysis.**

| study | Treatment arm | Control arm | Design | ORR, (n) | mPFS, HR (95%CI) | mOS, HR (95%CI) |
| --- | --- | --- | --- | --- | --- | --- |
| CheckMate-227 part1, 2022 | Nivolumab (3mg/kg Q2W) + Ipilimumab (1mg/kg Q6W) | chemotherapy | Phase 3 | 195/583 vs 162/583 | 0.79 (0.69-0.90) | 0.74 (0.65-0.84) |
| CheckMate-9LA, 2021 | Nivolumab (360mg Q3W) + Ipilimumab (1mg/kg Q6W) + chemo (2 cycles) | chemotherapy | Phase 3 | 137/361 vs 91/358 | 0.67 (0.56-0.79) | 0.72 (0.61-0.86) |
| MYSTIC, 2020 | Durvalumab (20mg/kg q4w) + Tremelimumab (1mg/kg q4w) | Platinum-base chemotherapy | Phase 3 | 56/163 vs 61/163 | 1.25 (1.05-1.49) | 0.94 (0.79-1.10) |
|  |  | Durvalumab (BTMB＞20） |  | 31/64 vs 23/77 | 0.76 (0.50-1.15) | 0.74 (0.48-1.11) |
|  |  | Durvalumab (BTMB＜20） |  | 34/204 vs 43/209 | 1.26 (1.02-1.57) | 1.22 (0.98-1.52) |
| The Lung-MAP, 2021 | Nivolumab (3mg/kg q2w) + Ipilimumab (1mg/kg q6w) | Nivolumab | Phase 3 | 23/124 vs 21/123 | 0.80 (0.61-1.03) | 0.87 (0.66-1.16) |
| KEYNOTE-598, 2022 | Pembrolizumab (200mg q3w) + Ipilimumab (1mg/kg q6w) | Pembrolizumab -placebo | Phase 3 | 47/284 vs 46/284 | 0.99 (0.81-1.21) | 1.05 (0.85-1.29) |
| ARCTIC, 2020 | Durvalumab (20mg/kg q4w) + Tremelimumab (1mg/kg q4w) | Soc | Phase 3 | 26/174 vs 8/118 | 0.77 (0.59-1.01) | 0.80 (0.61-1.05) |
|  |  | Durvalumab |  | 26/174 vs 18/117 | 0.87 (0.68-1.12) | 0.98 (0.74-1.30) |
|  |  | Tremelimumab |  | 26/174 vs 4/60 | 0.67 (0.49-0.92) | 0.78 (0.56-1.11) |
| CITYSCAPE, 2022 | Tiragolumab (600mg q3w) + Atezolizumab (1200mg q3w) | Atezolizumab -Placebo | Phase 2 | 26/67 vs 14/68 | 0.62 (0.42-0.91) | 0.69 (0.44-1.07) |
| NEPTUNE，2022 | Durvalumab (20mg/kg q4w) + Tremelimumab (1mg/kg q4w) | Soc (Global)^a^ | Phase 3 | 106/410 vs 172/412 | 1.08(0.92-1.25) | 1.02 (0.87-1.19) |
|  |  | Soc (China)^b^ |  | 28/78 vs 32/82 | 0.95(0.66-1.36) | 0.70 (0.48-1.02) |
| POSEIDON, 2022 | Durvalumab (1500mg q3w) + Tremelimumab (75mg q3w) + Soc | Soc | Phase 3 | 154/335 vs 110/332 | 0.72(0.6-0.86) | 0.77(0.65-0.92) |
|  |  | Durvalumab +Soc |  | 154/335 vs 160/335 | N/R | N/R |

Soc, Standard of care; OS, overall survival; PFS, progression-free survival; ORR, objective response rate; BTMB, blood tumor mutational burden

a, participants in Global cohort; b, participants in China cohort

**Supplementary Table 2. Stratification of safety outcomes measures considered in this pooled analysis.**

| study | Treatment arm | Control arm | All TRAE | TRAEs G3-5 (n) | DR, (n) |
| --- | --- | --- | --- | --- | --- |
| CheckMate-227 part1, 2022 | Nivolumab + Ipilimumab | chemotherapy | 442/576 vs 469/570 | 189/576 vs 205/570 | 104/576 vs 53/570 |
| CheckMate-9LA, 2021 | Nivolumab + Ipilimumab +chemotherapy | chemotherapy | 328/358 vs 306/349 | 173/358 vs 132/349 | 61/358 vs 21/349 |
| MYSTIC, 2020 | Durvalumab + Tremelimumab | chemotherapy | 223/371 vs 292/352 | 85/371 vs 119/352 | 49/371 vs 33/352 |
|  |  | Durvalumab (BTMB＞20） | 39/64 vs 51/77 | 10/64 vs 18/77 | 8/64 vs 3/77 |
|  |  | Durvalumab (BTMB＜20） | N/R | N/R | N/R |
| The Lung-MAP, 2021 | Nivolumab + Ipilimumab | Nivolumab | N/R | 49/124 vs 21/123 | N/R |
| KEYNOTE-598, 2022 | Pembrolizumab + Ipilimumab | Pem-placebo | 272/282 vs 263/281 | 176/282 vs 140/281 | 87/282 vs 13/281 |
| ARCTIC, 2020 | Durvalumab + Tremelimumab | Soc | 160/173 vs 105/110 | 74/173 vs 57/110 | 24/173 vs 9/110 |
|  |  | Durvalumab | 160/173 vs 109/117 | 74/173 vs 43/117 | 24/173 vs 2/117 |
|  |  | Tremelimumab | 160/173 vs 51/60 | 74/173 vs 25/60 | 24/173 vs 12/60 |
| CITYSCAPE, 2022 | Tiragolumab + Atezolizumab | Atezolizumab | 66/67 vs 66/68 | 35/67 vs 27/68 | 10/67 vs 9/68 |
| NEPTUNE，2022 | Durvalumab + Tremelimumab | Soc (Global) | 328/410 vs 329/413 | 193/410 vs 112/399 | N/R |
|  |  | Soc (China) | 50/78 vs 62/82 | 32/77 vs 22/78 | N/R |
| POSEIDON, 2022 | Durvalumab + Tremelimumab+ Soc | Soc | 251/338 vs 285/337 | 146/330 vs 117333 | N/R |
|  |  | Durvalumab +Soc | 251/338 vs 265/338 | 146/330 vs 134/334 | N/R |

Soc, Standard of care; TRAEs: treatment related adverse events; DR, (TRAEs led to) discontinued rate; N/R, not reported; BTMB, blood tumor mutational burden

**Supplementary Table 3. Safety evaluated between dual immunotherapy and chemotherapy.**

| Treatment related adverse events (TRAEs) | No. of trails | No. of patients (Events/Enrolled) | | RR (95%CI) | p |
| --- | --- | --- | --- | --- | --- |
|  |  | Dual immunotherapy | chemotherapy |  |  |
| Any grade | 4 | 1479/1966 | 1547/1876 | 0.89 (0.79-1.00) | 0.05 |
| Grade3-5 | 4 | 486/1478 | 496/1381 | 0.85 (0.62-1.16) | 0.31 |
| Serious | 6 | 725/2295 | 451/2191 | 1.55 (1.37-1.75) | ＜0.001 |
| Lead to discontinuation | 4 | 238/1478 | 116/1381 | 1.91 (1.44-2.54) | ＜0.0001 |
| Lead to death | 4 | 22/1478 | 15/1381 | 1.42 (0.74-2.74) | 0.29 |

**Supplementary Table 4. Safety evaluated between dual immunotherapy and ICIs monotherapy.**

|  | No. of trails | No. of patients (Events/Enrolled) | | RR (95%CI) | p |
| --- | --- | --- | --- | --- | --- |
|  |  | Dual immunotherapy | ICIs alone |  |  |
| Treatment related adverse events (TRAEs) | | | | | |
| Any grade | 4 | 766/1066 | 622/895 | 1.04 (1.00-1.07) | 0.03 |
| Grade3-5 | 5 | 421/1190 | 292/1018 | 1.29 (1.15-1.44) | ＜0.001 |
| Serious | 4 | 291/1068 | 172/895 | 1.49 (1.03-2.16) | 0.03 |
| Lead to discontinuation | 4 | 194/1066 | 53/945 | 2.86 (1.42-5.75) | 0.003 |
| Lead to death | 4 | 11/908 | 5/737 | 1.47 (0.40-5.37) | 0.56 |
| Immune related adverse events (irAEs) | | | | | |
| Any grade | 4 | 354/844 | 246/841 | 1.48 (1.14-1.93) | 0.003 |
| Grade 3-5 | 2 | 101/653 | 22/650 | 4.90 (1.41-17.01) | 0.01 |
